# The influence of the molecular packing on the room temperature phosphorescence of purely organic luminogens

**DOI:** 10.1038/s41467-018-03236-6

**Published:** 2018-02-26

**Authors:** Jie Yang, Xu Zhen, Bin Wang, Xuming Gao, Zichun Ren, Jiaqiang Wang, Yujun Xie, Jianrong Li, Qian Peng, Kanyi Pu, Zhen Li

**Affiliations:** 1 Department of Chemistry, Wuhan University, Wuhan, 430072 China; 2 School of Chemical and Biomedical Engineering Nanyang Technological University, Singapore, 637457 Singapore; 3 Department of Chemistry and Chemical Engineering, Beijing University of Technology, Beijing, 100124 China; 4 Key Laboratory of Organic Solids, Beijing National Laboratory for Molecular Science (BNLMS), Institute of Chemistry, Chinese Academy of Sciences, Beijing, 100190 China

## Abstract

Organic luminogens with persistent room temperature phosphorescence (RTP) have attracted great attention for their wide applications in optoelectronic devices and bioimaging. However, these materials are still very scarce, partially due to the unclear mechanism and lack of designing guidelines. Herein we develop seven 10-phenyl-10H-phenothiazine-5,5-dioxide-based derivatives, reveal their different RTP properties and underlying mechanism, and exploit their potential imaging applications. Coupled with the preliminary theoretical calculations, it is found that strong *π–π* interactions in solid state can promote the persistent RTP. Particularly, CS-CF_3_ shows the unique photo-induced phosphorescence in response to the changes in molecular packing, further confirming the key influence of the molecular packing on the RTP property. Furthermore, CS-F with its long RTP lifetime could be utilized for real-time excitation-free phosphorescent imaging in living mice. Thus, our study paves the way for the development of persistent RTP materials, in both the practical applications and the inherent mechanism.

## Abstract (web summary)

Organic luminogens with persistent room temperature phosphorescence will find wide applications in optoelectronic devices and bioimaging, but they are still scarce. Here, the authors synthesize seven organic luminogens and investigate their different properties and potential imaging applications.

## Introduction

Luminogens with room temperature phosphorescence (RTP) have attracted great attention for their full utilization of the excited state energy and long lifetime^1–14^. However, most of the RTP systems contain noble metals, which might suffer some intrinsic problems, including high cost, potential toxicity, and instability in aqueous environments. The alternative metal-free phosphors were seldom reported, especially those with persistent RTP effect. Recently, Kabe and Adachi have developed a purely organic host–guest doping system with unexpected but excited long persistent luminescence, continuing for more than 1 h at room temperature^15^. This could be even comparable to the most outstanding inorganic phosphors and has fully displayed the vast development prospect of purely organic RTP luminogens^16^.

Generally, unlike the relative complexity with the very careful control of dosage concentration for the host–guest doping system, utilization of organic RTP luminogens with single component should be more convenient, in addition to the corresponding easy exploration of inherent mechanisms. Thanks to the enthusiasm of scientists, enumerable pure organic single-component RTP systems have been developed, along with the proposed mechanisms^17,18^. For example, Huang and colleagues^19,20,]^ proposed that H-aggregation stabilized the triplet excitons through enhancing intersystem crossing process, in pursuit of ultralong phosphorescence at room temperature. Tang and colleagues^21,22,]^ suggested the crystallization-induced phosphorescence mechanism might be mainly responsible for the RTP character because of its effective inhibition of non-radiative decays. Chi and colleagues^23^ identified the key role of the intermolecular electronic coupling for achieving efficient persistent RTP. Based on these excellent pioneering works, systematical investigations are still needed to well understand the origin of persistent RTP.

According to the previous works, there are two main prerequisites that should be satisfied: the functional groups favoring *n*–π* transitions and the special packing in the solid state for the stabilization of the excited triplet state^2^. Relatively, the former is easier to be realized through the rational molecular design. The constructing blocks of sulfonyldibenzene and carbazole groups are two star ones to achieve the good communication between singlet and triplet states, because the existence of O or N atoms with lone pair electrons is capable of promoting *n*–π* transitions to populate triplet excitons (Supplementary Fig. 1)^3–14,17–30^. Here, we integrate 9-phenyl-9H-carbazole and sulfonyldibenzene groups together, and design a series of 10-phenyl-10H-phenothiazine 5,5-dioxide derivatives (CS-CH_3_O, CS-CH_3_, CS-H, CS-Br, CS-Cl, and CS-F) (Supplementary Fig. 2), with the aim to carefully investigate the structure–property relationship. Excitedly, accompanying with the adjustment of the substituent groups on the 10-phenyl ring from a methoxyl or methyl group to the hydrogen atom, then to the bromine, chlorine, or fluorine atoms, the RTP lifetimes in crystals of the corresponding luminogens increase from 88 ms (millisecond) (CS-CH_3_O) and 96 ms (CS-CH_3_) to 188 ms (CS-H), then to 268 ms (CS-Br), 256 ms (CS-Cl) and 410 ms (CS-F). Seemingly, the introduction of the electron-withdrawing substituents would be beneficial to the *π–π* interactions, which could stabilize the excited triplet state accompanying with the ultralong RTP effect in this system. Then, another substituent, the trifluoromethyl group, with even stronger withdrawing ability is introduced to yield CS-CF_3_. Although the longer RTP lifetime could not be observed, CS-CF_3_ crystal is found to possess another interesting character of reversible photo-induced RTP, which also should be originated from the reversible changes of molecular packing. Thus, the subtle change in the molecular structure does affect the molecular packing in crystal, resulting in the different RTP behaviors.

## Results

### RTP phenomena and the proposed mechanism

**Fig. 1:**
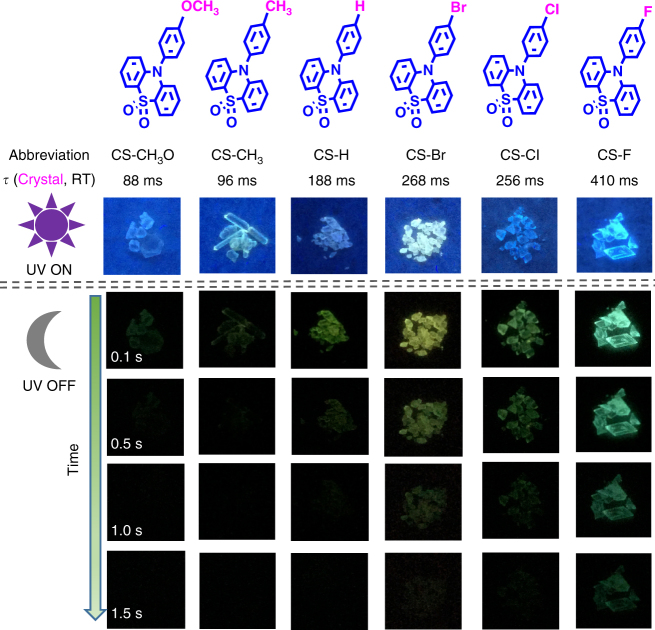
The room temperature phosphorescence (RTP) behavior of the six target compounds. The molecular structures of the six compounds of CS-CH_3_O, CS-CH_3_, CS-H, CS-Br, CS-Cl, and CS-F, and their corresponding phosphorescence lifetimes in crystals at room temperature (RT). The photographs were taken at different times, before and after turning off the 365 nm UV irritation under ambient conditions

**Fig. 2:**
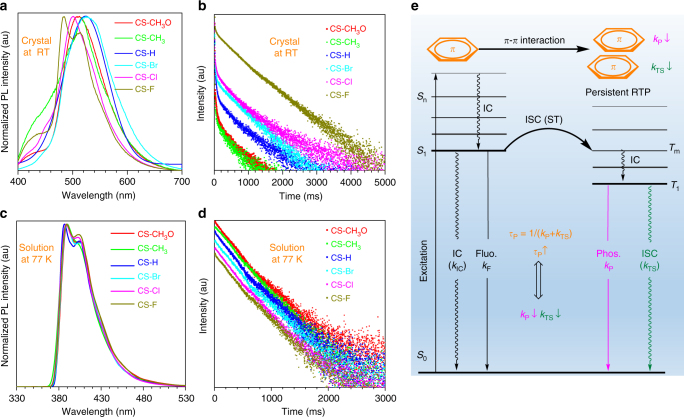
The phosphorescence spectra and corresponding time-resolved decay curves for the six compounds. **a** The normalized room temperature phosphorescence spectra of CS-CH_3_O, CS-CH_3_, CS-H, CS-Br, CS-Cl, and CS-F in crystal state. **b** Time-resolved PL-decay curves for their room temperature phosphorescence in crystal state. **c** The normalized low temperature (77 K) phosphorescence spectra in dichloromethane solution, concentration: 10^−5^ M. **d** Time-resolved PL-decay curves for their low temperature phosphorescence in dichloromethane solution, concentration: 10^−5^ M. **e** The proposed mechanism for organic persistent RTP: the strong *π–π* interaction could decrease the radiative transition (*k*_P_) and non-radiative transition (*k*_TS_) from *T*_1_ to *S*_0_ state, thus achieving the persistent room temperature phosphorescence

CS-CH_3_O, CS-CH_3_, CS-H, CS-Br, CS-Cl, CS-F, and CS-CF_3_ were facilely prepared through C–N coupling reactions, followed by the oxidation reaction with the aid of hydrogen peroxide (Supplementary Fig. 3). Upon the illumination of a 365 nm ultraviolet (UV) lamp, their as-prepared powders and crystals show blue or green emissions (Fig. 1). After stopping the photo-excitation, green or sky blue RTP emissions lasting for more than 1.5 s could be visually seen for CS-H, CS-Br, CS-Cl, and CS-F, while those of CS-CH_3_O and CS-CH_3_ would die out quickly. In the powder X-ray diffraction (PXRD) spectra of their as-prepared samples (Supplementary Fig. 4), CS-H, CS-Br, CS-Cl, and CS-F exhibit more sharp peaks than those of CS-CH_3_O and CS-CH_3_, indicating their better crystallinity. Their room temperature fluorescence, phosphorescence spectra, and the corresponding lifetimes in powder and crystal states were measured (Fig. 2, Supplementary Figs. 5–8, Supplementary Table 1). Particularly, the lifetimes of their room temperature phosphorescence in crystal state are much different from each other, ranging from 88 ms (CS-CH_3_O) and 96 ms (CS-CH_3_) to 188 ms (CS-H), then to 268 ms (CS-Br), 256 ms (CS-Cl), and 410 ms (CS-F), demonstrating some relation to the different substituent groups on the 10-phenyl ring.

In order to figure out the origin of the different RTP effect for these six compounds, their UV–visible absorption spectra at room temperature, low temperature phosphorescence, and the corresponding lifetimes in dichloromethane solutions were measured. As shown in Supplementary Fig. 9a, they share the similar UV–visible absorption spectra in solutions with three absorption peaks at about 275, 300, and 330 nm. Also, they all present two emission peaks at about 390 and 405 nm in their low temperature phosphorescence, with moderate differences of their corresponding lifetimes, ranging from 222 to 268 ms. These results are totally different from their photoluminescence (PL) behaviors in solid state, that is, the RTP spectra and the corresponding phosphorescence lifetimes are much different from each other (Fig. 2). These experimental results displayed that the packing modes, rather than the molecular electronic structures, should mainly account for the different RTP behaviors in solid state.

Generally, most of pure organic molecules are fluorophores with very short-lived singlet exciton for fluorescence. Only a small part could realize the transition from the excited singlet state to the triplet one, which then decays from the *T*_1_ state to the *S*_0_ state through the phosphorescent process with the lifetime short than 10 ms. In order to obtain the ultralong phosphorescence, a stable excited triplet state (*T*_1_*) should be formed, which could either lower energy levels to decrease the radiative decay rate or restrain the molecular motion to result in the diminished non-radiative decay rate (Fig. 2e). In this system, the stable *T*_1_* state must have been formed in view of the observed persistent RTP effect.

### Molecular packing

**Fig. 3:**
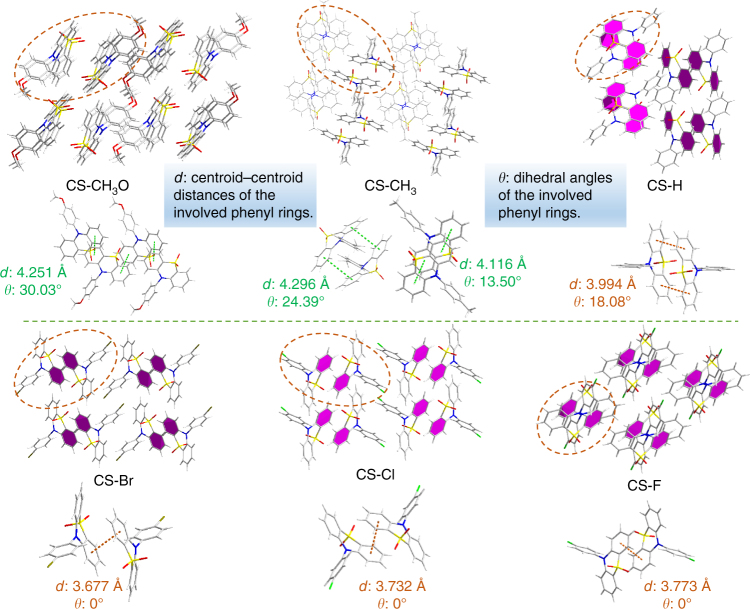
Single-crystal structures of the six target compounds. Entire and local packing modes of the crystals for CS-CH_3_O, CS-CH_3_, CS-H, CS-Br, CS-Cl, and CS-F: the local packing pictures were selected from the parts in cycles of corresponding entire ones, in which the centroid–centroid distances and the dihedral angles of the involved phenyl rings are listed and the phenyl rings involved in the stronger *π–π* interactions in entire are labeled by pink color

In order to find out what kind of packing mode could lead to the stabilized *T*_1_* state in this system and make a deep insight into the persistent RTP, we cultured the single crystals of these six compounds (Supplementary Table 2). Figure 3 shows the entire and local packing modes of these crystals, which are different from each other according to the subtly different substituent groups on the 10-phenyl ring. Analyzing carefully, the compounds of CS-Br, CS-Cl, and CS-F, with electron-withdrawing groups, show much stronger *π–π* stackings with the involved two phenyl rings paralleling to each other. And the centroid–centroid distances between them are much shorter (ranging from 3.677 to 3.732 and 3.773 Å). However, the *π–π* interactions between the adjacent phenyl rings are too weak to be considered for the compounds with electron-donating substituents. For CS-CH_3_O, there are four molecules in a minimum repeat unit, and the centroid–centroid distances between two adjacent benzenes are 4.251 Å, while their dihedral angles are all about 30^o^. In CS-CH_3_, there are two different kinds of molecular configurations of CS-CH_3_(a) and CS-CH_3_(b); a is in thin bonds and b is in thick bonds. Every two of the same configurations are coupled together, with the distance and dihedral angle between two adjacent benzenes of 4.296 Å and 24.39^o^ in CS-CH_3_(a) respectively, while they are 4.116 Å and 13.50^o^ for CS-CH_3_(b). As for CS-H, it shows a medium centroid distance and dihedral angel between the adjacent phenyl rings, namely 3.994 Å and 18.08^o^ respectively. These crystal data demonstrate that the phosphors with acceptor substituents (CS-Br, CS-Cl, and CS-F) show much enhanced *π–π* interactions, rather than CS-H without substituent and those (CS-CH_3_O and CS-CH_3_) with donor substituents, in good accordance to the decreasing tendency in their RTP lifetimes for crystals. Thus, the packing mode with strong *π–π* interactions could be considered as the main origin for the formation of the stabilized *T*_1_* state, accompanying with the persistent RTP effect. This phenomenon could be termed as one of Molecular Uniting Set Identified Characteristic (MUSIC).

Further on, we took two molecules in our system as examples to study their aggregate mode: one was CS-CH_3_O with the shortest RTP lifetime of 88 ms and another was CS-F with the longest RTP lifetime up to 410 ms. Also, a famous RTP luminogen of DEOPh with H-aggregation was studied for comparison. According to the experimental results, it was clear that CS-CH_3_O and CS-F were all J-aggregates for the bathochromic-shifted absorption from solution to aggregation, while DEOPh was H-aggregate for the hypochromatic-shifted absorption from solution to aggregation (Supplementary Fig. 10). Thus, it is the π–π stacking rather than H-aggregation that should be mainly responsible for the RTP effect in our system. On the other hand, the absorptions of both CS-CH_3_O and CS-F were equally affected by aggregation, but they show marked different RTP responses in their crystal forms, and thus it was concluded that the aggregates in ground state were not the main origin, at least not the sole origin responsible for their different RTP behaviors in the crystals.

Also, the strength of intermolecular interactions (C-H…*π*, C-H…O, C-H…N, C-H…F, and so on) should also be taken into consideration for the persistent RTP effect, because of its effective suppression to the non-radiative decay^20,31,32^. For compounds CS-F, its longest phosphorescence lifetime (410 ms) in crystal might mainly be originated from the enhancement of intermolecular interactions, although their centroid–centroid distance (3.773 Å) in *π–π* stacking is a little longer than those of CS-Br (3.677 Å) and CS-Cl (3.732 Å). The raised PXRD peaks (Supplementary Fig. 4) and melting points from 224 °C (CS-Br) to 242 °C (CS-Cl) and 247 °C (CS-F) could also well suggest the enhancement of intermolecular interactions from CS-Br and CS-Cl to CS-F^33^. Thus, the synergistic effect of strong *π–π* stacking and efficient intermolecular interactions promotes the persistent RTP character in this system.

### Effect of *π–π* interaction on excited state

**Fig. 4:**
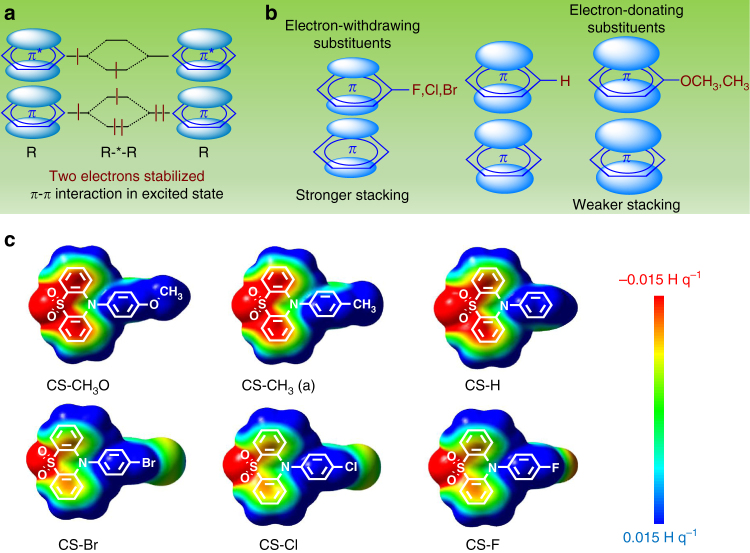
The influence of *π–π* interactions on the electron redistribution and RTP behavior. **a** The orbital interaction of *π–π* stacking in excited state: the electrons would redistribute in new orbitals for the *π–π* interactions, then three electrons are stabilized and one electron is destabilized, and thus the net stabilization is two electrons. **b** Depiction of the electrostatic model of substituent effects on *π–π* interactions from Hunter–Sanders model: electron-withdrawing substituents could enhance *π–π* interactions by decreasing the *π*-electron density of the substituted *π*-system and relieving the *π–π* repulsion, while electron-donating substituents hinder *π–π* stacking through the opposite mechanism. **c** Difference electrostatic potential (ESP) analysis of isolated CS-CH_3_O, CS-CH_3_ (a), CS-H, CS-Br, CS-Cl, and CS-F. The potential energy range is −0.015 to 0.015 H q^-1^ for all surfaces shown, red indicates areas with dense electron density, yellow for normal, while blue areas suggest less electron density

In order to reveal the internal mechanism of why strong *π–π* interactions could stabilize the excited triplet state, the research on molecular orbitals of molecules with the strong π–π stacking was carried out (Supplementary Table 3). As shown in Fig. 4a, when two *π* systems are colliding, the main electronic interactions will involve their highest occupied molecular orbital (HOMO) and lowest unoccupied molecular orbital (LUMO)^34^. According to the perturbation theory, the HOMOs of two *π* systems interact with each other, then produce two new HOMO orbitals, while the same process occurs for their LUMO orbitals. The newly formed HOMOs and LUMOs are split in energy relative to the original HOMOs and LUMOs, one of the new orbital is lower in energy than the original one, while another is higher. Thus, when a photon, absorbed by one *π* system, jumps to the excited state, the electrons would redistribute in the new excited state for the *π–π* interactions. Then three electrons are stabilized (two on the lower energy HOMO, one on the lower energy LUMO), while only one electron is destabilized in the higher energy HOMO. Thus, a new excited state with lower energy could be achieved for the *π–π* interactions. On the other hand, no vibrational energy sub-levels exist for this excited state and the corresponding ground state of vertical energy transition, which could inhibit the non-radiative transition. Thus, it is proposed that the *π–π* interaction could lead to the formation of a new particular excited state with slow radiative decay and non-radiative decay rate, just like *T*_1_* state. Then, the slower radiative decay (*k*_P_) and restricted non-radiative decay (*k*_TS_) from *T*_1_ to *S*_0_ could both stabilize the excited triplet state and lead to the longer phosphorescence lifetime (*τ*_P_); see Eq. (1) below:

1 $${\mathrm{\tau }}_{\mathrm{P}} = 1{\mathrm{/}}\left( {k_{\mathrm{P}} + k_{{\mathrm{TS}}}} \right).$$

To obtain a deep insight into the RTP process, the energy levels of excited states were calculated for the isolated molecules and coupled units with *π–π* interaction derived from the single-crystal structures (Supplementary Table 4)^35^. All of the six compounds give the similar *T*_1_ energies in the range of 3.8256–3.9760 eV, just like the case observed in their low temperature phosphorescence, indicating the reliability of theory calculations to some extent. When two molecules couple together, the energies of *T*_1_* for coupled units show varying degrees of decline, in comparison with that of *T*_1_ for isolated molecules. For CS-CH_3_O and CS-CH_3_ with electron-donating substituents and weak *π*–*π* interactions, the declines in the coupled ones are much less, ranging from 0.0281 to 0.0573 eV. On the contrary, the compounds of CS-Br, CS-Cl, and CS-F with electron-withdrawing substituents and stronger *π–π* interactions exhibit much larger declines from the *T*_1_ of isolated molecules to *T*_1_* of coupled units, ranging from 0.0865 to 0.1186 eV. These calculated results could well certify the strong *π–π* interactions as the main origin to lower the energy and reducing the radiative decay from *T*_1_* to *S*_0_ state, thus leading to the promotion of RTP lifetimes. Also, calculations on energy levels of these compounds in the crystalline state were carried out by combined quantum mechanics and molecular mechanics (QM/MM) method (Supplementary Figs. 11–16, Supplementary Table 4–6). As shown in Supplementary Table 4, the energies for the central monomer molecules in crystalline states were similar to those of the isolated molecules, while those in coupled units were much different, which could prove the great effect on energy levels for the formation of coupled ones once more. The calculations in gas state were also carried out (Supplementary Figs. 11–16, Supplementary Table 5), and the optimized structures were similar to those in crystals.

### Effect of electronic effect on *π–π* interactions

It is interesting that, in this system, the compounds with electron-withdrawing substituents would favor the stronger *π–π* interactions, while others with electron-donating substituents not. Considering the close relationship between molecular packing and property, it would be of great importance to make clear how the substituents affect the *π–π* interaction. Actually, in 1990, the pioneering works of Hunter and Sanders^36^ have provided an intuitive physical model of the substituent effect on the *π–π* interaction^37^. They proposed that the strength and preferred orientations of *π–π* interactions between adjacent aromatic rings could be understood and predicted based on a simple electrostatic model. As shown in Fig. 4b, the electron-withdrawing substituents (–Br, –Cl, –F, and so on) could enhance *π–π* interactions by decreasing the *π*-electron density of the substituted *π*-system and relieving the *π–π* repulsion between the two involved rings. On the other hand, the electron-donating substituent (–OCH_3_, –CH_3_, and so on) would lead to the dense *π*-electron density, and hinder *π–π* interactions. Just according to the Hunter–Sanders model, the above explanation could be given for the preferred molecular packing in crystals of these six compounds, which could guide the molecular design of persistent RTP materials to some extent.

Furthermore, the calculations on the electrostatic potential (ESP) were carried out, which have often been used to explore the origin for different strengths of *π–π* interactions^38,39^. The ESP is the electrostatic interaction that a positive test charge would experience at that position at a given point in space in the vicinity of a molecule, and it could reflect the difference of the electron density distribution. As presented in Fig. 4c and Supplementary Table 7, the potential energy range is from −0.015 to 0.015 H q^−1^ for all surfaces demonstrated; the red color shows areas with the dense electron density, yellow for normal, while blue areas suggest less electron density. The detailed comparison of the electrostatic potential surfaces of these six compounds suggests that CS-CH_3_O and CS-CH_3_ with electron-donating substituents present the dense electron density (in red color) on the phenyl rings involved in the *π–π* interactions, then CS-H without substituent. As for CS-Br, CS-Cl, and CS-F with electron-withdrawing substituents, their electron densities of the corresponding phenyl rings are largely reduced (in yellow color) for the induction effect of Br, Cl, and F atoms. The reduced electron density on the face of rings would relieve the *π–π* repulsion between the two involved rings, thus resulting in the shorter *π–π* distance and stronger *π–π* interaction.

### Photo-induced phosphorescence effect

**Fig. 5:**
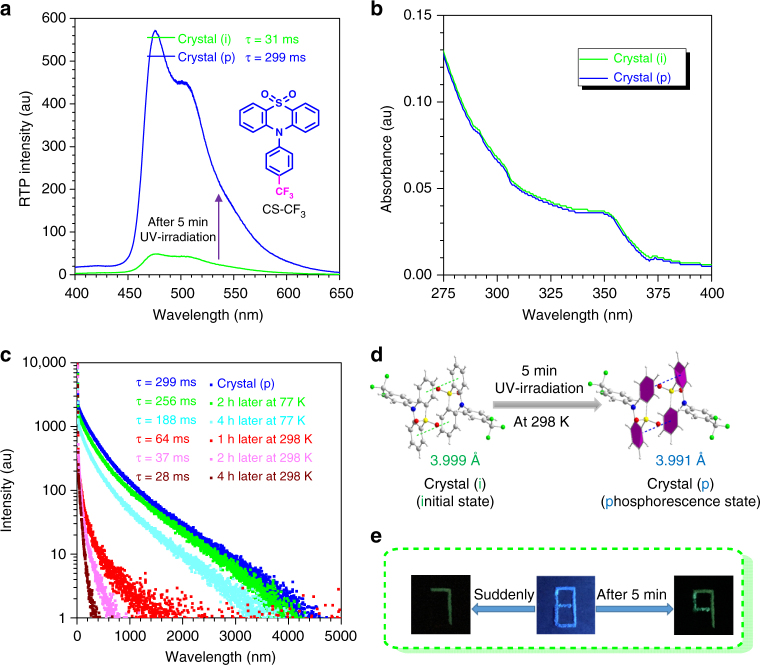
The photo-induced phosphorescence property of CS-CF_3_. **a** The room temperature phosphorescence spectra of CS-CF_3_ crystal before and after 365 nm UV irradiation for 3 min. **b** The UV–visible spectra of the CS-CF_3_ crystal before and after UV irradiation for 5 min. **c** Time-resolved PL-decay curves for the room temperature phosphorescence of CS-CF_3_ crystal under 5 min 365 nm UV irradiation and after 1 or 2 or 4 h of standing at 77 K or 298 K. **d** The crystal structures of coupled CS-CF_3_ before and after UV irradiation for 5 min. **e** Double security protection applications by using three kinds of components of CS-CF_3_, CS-F and (4-methoxyphenyl)(phenyl)methanone. Under 365 nm UV irradiation, it presents blue pattern 8; then switching off the UV light suddenly, it turns to green pattern 7; if the UV irradiation could be kept for about 5 min, it would appear green pattern 9 after turning off the UV light

According to the above results, it could be proposed that the introduction of stronger electron-withdrawing substituent on the 10-phenyl ring would relieve the *π–π* repulsion and lead to the effective *π–π* interaction, thus resulting in the stronger persistent RTP effect. To further check this point, the trifluoromethyl group with even stronger electron-withdrawing ability was introduced to yield CS-CF_3_ (Fig. 5, Supplementary Fig. 17). Unexpectedly, the as-prepared CS-CF_3_ powder did not show visible RTP, and even no RTP was observed in its single crystal, totally out of the rule summarized from the above six compounds. Excitedly, it turned to show obvious persistent RTP effect upon UV irradiation for 5 min, accompanying with increased RTP lifetime to 299 ms (Fig. 5a). Then it would return to the initial state after several hours of standing under natural conditions at room temperature. This has never been reported before, and could be termed as photo-induced room temperature phosphorescence. Thus, we monitored the excitation and relaxation procedure of RTP intensity and lifetimes, and it was found that transition from crystal (i) to crystal (p) was finished within 4 min under the UV irradiation at 365 nm (Supplementary Fig. 18). Over 4 min, the RTP intensity and lifetime would nearly not change. After stopping the UV irradiation, they both decreased with standing time, then returned to the initial state after several hours (Supplementary Fig. 19).

To explore the origin of this unique property, the UV–visible absorptions of CS-CF_3_ crystal before and after 5 min of UV irradiation were measured but without obvious change, indicating no molecular structure change during this process (Fig. 5b). Similarly, the phosphorescence excitation peaks retained before and after UV irradiation at 365 nm for 5 min, and just the enhanced excitation intensity could be observed (Supplementary Fig. 20). As reported in the literatures, for some molecules, there were some molecular motions present in their single crystals under UV irradiation, which could lead to big changes of the photophysical properties, such as the fluorescence or morphology change, but not concerning the phosphorescent change^40–44^.

In order to make clear whether there are molecular motions in CS-CF_3_ crystal under UV light, we measured the single-crystal structures of CS-CF_3_ before and after UV irradiation for 5 min. As shown in Fig. 5d, Supplementary Table 8 and Supplementary Fig. 21, the distance between two adjacent phenyl rings in the coupled CS-CF_3_ changed from 3.999 Å at the initial state to 3.991 Å after UV irradiation for 5 min in the phosphorescence state. Although the change of the *π–π* distance in crystal between the initial and phosphorescence states is too small and nearly ignorable, the apparent RTP process could confirm the significant variation. In the process of UV stimulation, enhanced *π–π* interactions might have been achieved, which led to the appearance of persistent RTP character. Considering the long time needed in the measurement of single crystal (about 1 h), the relaxation would happen from the phosphorescence state to initial one, and thus the minor changes in the crystal packing might be reasonable. On the other hand, the X-ray might make some influences on the molecular packing under test conditions. 

Furthermore, based on the model in Fig. 4a, molecules tend to form enhanced *π–π* interactions for the lower energy effect in the excited state, and thus when CS-CF_3_ in crystal was excited under UV irradiation, the molecular motion processed. After a period of time, the crystal moved to a more stable packing status in the excited state, in which the *π–π* interactions were enhanced, thus achieving the ultralong RTP character. However, about 2 h later without the excitation of UV irradiation, the phosphorescence crystal would return to the initial state for its more stable ground state. Thus, the reversible photo-induced phosphorescence could be certainly ascribed to the reversible change of crystal packing. As for the as-prepared CS-CF_3_ powder, no required *π–π* interactions could be formed even after a long time of UV irradiation for the irregular molecular arrangement (Supplementary Fig. 22). Thus, no significant RTP character could be observed after the UV irradiation.

To further confirm the change of molecular packing as the main origin for its photo-induced phosphorescence effect, the control experiments under low temperature (i.e., 77 K) were conducted^45^. As shown in Supplementary Fig. 23, no obvious changes including RTP intensity and lifetime could be observed before and after 5 min of UV irradiation on the initial CS-CF_3_ crystal at 77 K. This should be ascribed to the effective inhibition effect on molecular motion under low temperature. Further on, the low temperature (i.e., 77 K) could restrict the decay for phosphorescence state to initial state of crystal CS-CF_3_: the RTP lifetime could retain at 256 ms after 2 h of standing at 77 K, while it decreased to just 37 ms at room temperature (Fig. 5c). Similarly, much less damping for RTP intensity was observed after 2 h of standing at 77 K in comparison with that at room temperature (Supplementary Fig. 24). Thus, these control experiments could certify significant influence of molecular motion and molecular packing on the unique photo-induced phosphorescence effect.

Given the interesting reversible photo-induced RTP characteristic of CS-CF_3_, it is a good candidate as double document security. As shown in Fig. 5e and Supplementary Fig. 25, the original pattern of 8 was made of three kinds of components, one was CS-CF_3_ with the characteristic of the reversible photo-induced phosphorescence, the second was CS-F with normally persistent RTP characteristic, and the last one was (4-methoxyphenyl)(phenyl)methanone without the RTP property. Once the pattern was excited under a 365 nm UV lamp, it presented a blue 8. Switching off the UV light suddenly, the green 7, encrypted by CS-F molecule with the ultralong RTP, could be readily visualized, while the other parts made of CS-CF_3_ and (4-methoxyphenyl)(phenyl)methanone lightness. However, if keeping the UV irradiation for about 5 min, the part of CS-CF_3_ would be turned to be RTP active. Then, switching off the UV source, a totally different pattern of a green 9 would arise and disappear gradually, which was the integration of CS-F and CS-CF_3_. Furthermore, this process is reversible and the persistent RTP pattern of green 7 could be recovered after about 2 h of standing without UV irradiation. This reversible process at room temperature without any other external stimulation makes CS-CF_3_ a promising candidate for double security protection material.

### In vivo phosphorescent imaging

**Fig. 6:**
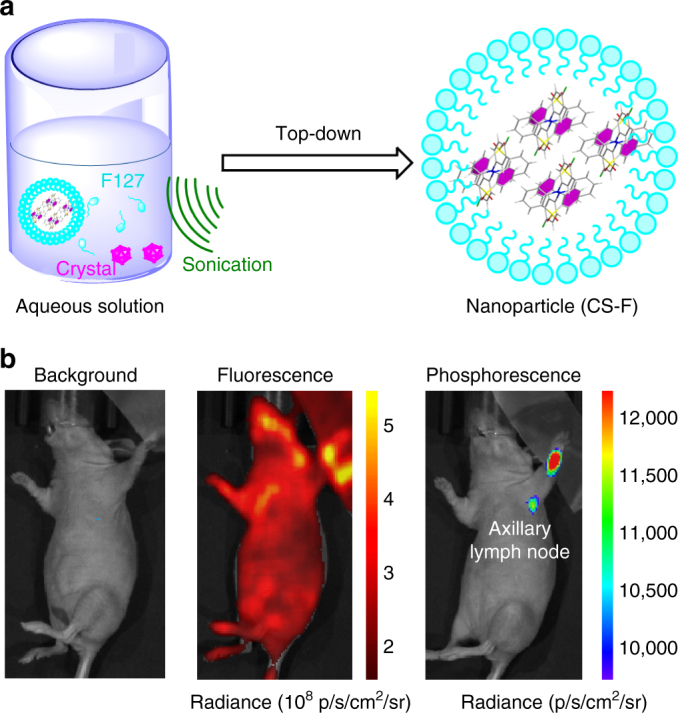
In vivo real-time excitation-free phosphorescent imaging of lymph nodes. **a** Top-down approach to synthesize the water-soluble nanoparticles of CS-F: the crystal was added to the aqueous solution of PEG-b-PPG-b-PEG (F127) under continuous sonication, then the aqueous solution was filtered through a 0.22 µm polyvinylidene fluoride (PVDF) (Millipore). **b** Ultralong phosphorescence and fluorescence imaging of lymph node in living mice 1 h after the intradermal injection of CS-F nanoparticles into the forepaw of mice

Furthermore, compounds CS-F and CS-CF_3_ were selected to test the potential applications in in vivo phosphorescent imaging. Top-down approach was utilized to prepare water-soluble organic nanoparticles based on CS-F or CS-CF_3_ with ultralong phosphorescence, which could take advantage of strong molecular packing of RTP luminogens to generate aggregates within nanoparticles (Fig. 6a and Supplementary Figs. 26–27)^10^. It was a little pity that photo-induced RTP effect could not be achieved in the CS-CF_3_ nanoparticles (Supplementary Figs. 28–30), partially due to the opaqueness of nanoparticles. Thus, particular attention was paid to the CS-F nanoparticles with the longer RTP lifetime. First, the solution of CS-F nanoparticles was irradiated with UV light for 1 min to activate nanoparticles, then the ultralong phosphorescence can be easily detected even at *t* = 10 s after removal of light source (Supplementary Figs. 29–30). Also, the ability of the CS-F nanoparticles for in vivo imaging was validated in living mice. CS-F (0.5 mg ml^−1^, 50 µl) were administered to the forepaws of living mice anesthetized using 2% isoflurane in oxygen via intradermal injection. At *t* = 1 h post injection, nanoparticles were activated by irradiation with 365 nm hand-held UV lamp for 1 min. Then the image was acquired at *t* = 10 s after removal of light source using IVIS living imaging system under the bioluminescence mode. As shown in Fig. 6b, the ultralong phosphorescence could be easily detected with RTP intensity up to 12,000 p s^−1^ cm^−2^ sr^−1^, comparable to the previous report about organic RTP luminogens^10^. For comparison, the in vivo fluorescence imaging of mice was also carried out (excitation: 430 ± 15 nm; emission: 600 ± 10 nm) and the background fluorescence was too strong to distinguish the signal of the CS-F nanoparticles. Thus, these results well demonstrated the potential of CS-F for real-time excitation-free in vivo phosphorescent imaging.

## Discussion

As revealed by our experimental data, the intermolecular *π–π* interaction plays an important role for the RTP effect. Actually, this viewpoint is applicable to other RTP systems in which the *π–π* distances in single crystals are found to have direct relationship with RTP lifetimes (Supplementary Figs. 31–32), further confirming the accuracy of the statement^21,46^. Based on our research and other successful cases reported so far, mainly three issues should be taken into consideration to design the persistent RTP materials.

First, the N, O atoms and so on with lone pair electrons should be introduced, which would contribute much to the *n*–π* transition and intersystem crossing, thus achieving the efficient RTP^7–28,47^.

Secondly, the strong *π–π* stacking in solid state should be considered as one of the main origin for the ultralong lifetime of RTP materials, since it could decrease the radiative decay rate (*k*_P_) or non-radiative decay rate (*k*_TS_) from *T*_1_ to *S*_0_ state, and lead to the longer RTP lifetime through the Eq. (1)^18,48,49^.

The third one is how to enhance the *π–π* interactions in solid state from the initial molecular design. There are mainly two ways: one is to relieve the *π–π* repulsion, while another is to increase the *π–π* attraction (Supplementary Fig. 33). In our system, the introduction of electron-withdrawing substituents (Br, Cl, F, and so on) decreases the *π*-electron density of the substituted *π*-system and relieves the *π–π* repulsion between the two involved rings, thus realizing the strong *π–π* stacking. Alternatively, the reduction of the steric hindrance is also an efficient way to relieve *π–π* repulsion, and thus the molecule with a planar conformation seems a good choice^19,46^. Then, the electrostatic interaction, dipole–dipole interaction an so on are considered as the main driving forces to enhance the *π–π* attraction with the dense *π–π* stacking. The Integration of the electron donor and acceptor in one molecule would be much beneficial to the intermolecular electrostatic interaction or dipole–dipole interaction and so on, possibly leading to the enhanced *π–π* attraction and strong *π–π* stacking^19,20,50^. Eventually, the strong *π–π* stacking in solid state might lead to the persistent RTP effect.

However, it is still not clear that either the aggregates in ground state or the formed excimer in excited state should be mainly responsible for the persistent RTP effect in this system. In the beginning, it is believed the triplet excimers have been formed in the crystals through the following scheme:

$$h\nu + {\mathrm{R}} \to {}^1{\mathrm{R}}^ \ast \to {}^1{\mathrm{E}}^ \ast \to {}^3{\mathrm{E}}^ \ast ,$$

in which R is the ground-state molecule, ^1^R* is the excited singlet state molecule, ^1^E* is the singlet excimer, ^3^E* is the triplet excimer. Then, the ^3^E* would lead to their ultralong RTP lifetimes. If it is, the phosphorescence excitation spectra should match the absorption spectra^51^. Unfortunately, the absorption and phosphorescence excitation spectra do not match each other as expected (Supplementary Fig. 34). The excitation spectra show a pronounced contribution to the phosphorescence emission from states that may exist at the onset of the absorption but seem to have very low oscillator strength to be observed. These can be aggregates. The triplet excimer mechanism therefore cannot be confirmed in this way. It cannot be simply discarded either as reabsoprtion/satuartion effects in crystals may be the cause of this mismatch.

However, the absorption spectrum of CS-CF_3_ crystal in Fig. 5b does not show significant differences upon irradiation, and this indicates that no new ground-state species are being formed. Once more, the phosphorescence excitation spectrum of CS-CF_3_ crystals is entirely different when compared with absorption spectra, and again shows a red-shifted absorption band at the onset of the absorption spectrum. This agrees with the mismatch between the absorption and excitation spectra observed in the other molecules, and indicates the underlying phenomena to explain the long-lived RTP should be the same. However, it is clear that some sort of molecular rearrangement is involved on the observation of long-lived RTP in CS-CF_3_ crystals (Fig. 5d) and upon irradiation, the CS-CF_3_ molecules have to rearrange themselves to form the species that gives origin to the long-lived phosphorescence. Such interaction does not occur in the non-irradiated molecules as the lifetime of the RTP is much shorter in the non-irradiated samples. Therefore, we can conclude that the irradiation in CS-CF_3_ molecules may create sandwich type species which give origin to the long-lived RTP. This is very similar to the formation of excimers; i.e., immediately upon irradiation the CS-CF_3_ molecules are locked in the excimer configuration and continue giving origin to the long-lived RTP, but once the irradiation is turned off and as time passes, the excimer configuration is being lost due to dissociation induced by thermal-assisted vibrations, and the long-lived RTP disappears over time after irradiation has been turned off. Hence, the scenario in CS-CF_3_ crystals seems to favor the formation of the excimer mechanism. However, with the data presented, it is not possible to completely discard the formation of ground-state species (aggregates) to explain the long-lived RTP. Thus, if we want to clarify the internal mechanism absolutely, more works should be done.

In summary, a series of 10-phenyl-10H-phenothiazine 5,5-dioxide derivatives have been synthesized, which demonstrate different phosphorescence properties at room temperature. Detailed investigation confirmed that the strong intermolecular interactions in solid state, inherited from the molecular electronic property and mainly determined by the molecular packing, accounted for their persistent RTP, exhibiting the effect of MUSIC. Excitedly, CS-CF_3_ crystal shows unique reversible photo-induced phosphorescence effect due to the change of the molecular packing under UV irradiation. Furthermore, CS-F can be used for real-time excitation-free phosphorescent imaging of lymph nodes in living mice.

## Methods

### In vivo afterglow imaging

Preparation of nanoparticles: The solids of CS-F or CS-CF_3_ (1 mg) were added to the aqueous solution of PEG-b-PPG-b-PEG (F127) (3.33 mg ml^−1^, 3 ml) under continuous sonication with a microtip-equipped probe sonicator (Branson, S-250D) for 10 min. Finally, the aqueous solution was filtered through a 0.22 µm polyvinylidene fluoride (PVDF) syringe-driven filter (Millipore).

Dynamic light scattering was performed on the Malvern Nano-ZS Particle Size. Ultraviolet–visible (UV–Vis) and fluorescence spectra were obtained using a SHIMADZU UV-3600 UV-VIS-NIR spectrophotometer and a RF-5301PC spectrofluorophotometer, respectively.

All animal studies were performed in compliance with the guidelines set by the Institutional Animal Care and Use Committee, Sing Health. Female nude mice were used for in vivo subcutaneous imaging and lymph node imaging. For subcutaneous in vivo optical imaging, mice were anesthetized using 2% isoflurane in oxygen, and then received two subcutaneous injections of organic nanoparticles or fluorescein (1.6 µM) into the dorsal areas of mice. After subcutaneous injection of nanoparticles, in situ activation of ultralong phosphorescence was conducted by irradiation with a 365 nm hand-held UV lamp for 1 min. The power density of the UV lamp was measured to be 10 mW cm^–2^ using a Thorlab PM100D power meter equipped with a S130VC power head with Si detector. It is lower than the maximum power exposure allowed for skin irradiation with 1 min of UV light (315 to 400 nm) (18 mW cm^–2^). The UV lamp was put 1 cm above the injection sites of RTP nanoparticles. Then, the images were acquired at *t *= 10 s after removal of light source using IVIS living imaging system. The signal acquisition was conducted for 10 s under the bioluminescence mode with open filter setting. During the imaging, the mice were warmed with a heating pad under continued isoflurane anesthesia. For comparison, the in vivo fluorescence imaging of mice was also carried out (excitation: 430 ± 15 nm; emission: 500 ± 10 nm).

### Data availability

The authors declare that the data supporting the findings of this study are available within the article and its Supplementary Information file. All data are available from the authors upon reasonable request.

## Electronic supplementary material

Supplementary Data 2

Supplementary Information

Description of Additional Supplementary Files

Supplementary Movie 1

Supplementary Movie 2

Supplementary Data 1
