# Supplementary material for: The influence of the molecular packing on the room temperature phosphorescence of purely organic luminogens
Source: Nat Commun. 2018 Feb 26;9:840. doi: 10.1038/s41467-018-03236-6 (PMC5826932; doi:10.1038/s41467-018-03236-6)
Supplement: Supplementary file 2 — Supplementary Information [file 41467_2018_3236_MOESM2_ESM.pdf]

The influence of the molecular packing on the room temperature phosphorescence of purely organic luminogens

Yang *et al.*

Supplementary Methods

^{1}H NMR and ^{13}C NMR spectra were recorded on a 300 MHz Varian Mercury or 400 MHz Bruker Advance III spectrometer using $CDCl_3$ as solvent. Mass spectra were conducted on a ZAB 3F-HF mass spectrophotometer. Elemental analyses of carbon, hydrogen, nitrogen and sulfur were measured on a Perkin-Elmer microanalyzer. UV-vis spectra were performed on a Shimadzu UV-2550. Photoluminescence spectra at room temperature and 77K were performed on a Hitachi F-4600 fluorescence spectrophotometer. Fluorescence decays were obtained using a spectrometer (FluoroLog-TCSPC) from HORIBA Instruments. The powder X-ray diffraction patterns were recorded by Rigaku MiniFlex 600 with an X-ray source of Cu Kα (λ = 1.5418 Å) at 25 ^{o}C at 40 KV and 15 mA at a scan rate of 10° (2θ)/min (scan range: 5-50°). The single-crystal X-ray diffraction data were collected in a Bruker Smart Apex CCD diffractometer. Melting points were recorder on SGW X-4B microscopic melting point meter.

The molecular structures in gas state were optimized at m062x/6-31g* level in the Gaussian 09 software. The aggregation effect was considered through the combined quantum mechanics and molecular mechanics (QM/MM) theory with two-layer ONIOM method implemented in the D.01 version of Gaussian 09 package. Our computational models were built from the single crystal structures without further optimization. The centroid monomer molecule of the cluster was chosen as QM region (high layer), whereas the remaining molecules were treated as MM region (low layer). The vertical excitation energies were obtained at the non-optimized configurations of the ground states. The m062x functional together with 6-31g* basis set was employed for the QM calculation. The universal force field (UFF) was used for the MM expressions. The electrostatic embedding scheme was adopted in the QM/MM calculations, incorporating the MM partial charges into the effective Hamiltonian of the QM part. The calculation based on isolated molecules and coupled units were obtained from single crystal structures and no further geometry optimizations were conducted in order to remain the specific molecular configurations and corresponding intermolecular locations. The vertical excitation energies were calculated by using TD-DFT calculation (m062x/6-31g*) for electronic excited singlet and triplet states.

Synthesis

General procedure for the synthesis of **CS-CH$_3$O, CS-CH$_3$, CS-H, CS-Br, CS-Cl, CS-F** and **CS-CF$_3$**.

CS-CH$_3$O: Phenothiazine (1.99 g, 10 mmol), 1-iodo-4-methoxybenzene (2.34 g, 12 mol), potassium tert-butoxide (1.68 g, 15 mmol), palladium acetate (0.11 g, 0.5 mmol) and tri-tert-butylphosphine solution (0.5 mL, 0.25 mmol) were dissolved in toluene (100 mL) in a Schlenk tube. The resultant mixture was refluxed for 12 hours under argon, then extracted with dichloromethane. The combined organic extracts were dried over anhydrous Na_2SO_4 and concentrated by rotary evaporation. The crude product was purified by column chromatography on silica gel using petroleum ether as eluent. Then the collected product was dissolved in dichloromethane (90 mL), acetic acid (45 mL) and H_2O_2 (2 mL). After reacting for another 24 hours at 60 ^{o}C, the reaction mixture was extracted with dichloromethane and further purified by column chromatography using petroleum ether/ethyl acetate (8:1 v/v) as eluent to afford a white solid in a yield of 80%. mp: 251 ^{o}C; ^{1}H-NMR (300 MHz, $CDCl_3$): δ 8.15-8.17 (d, 2H), 7.36-7.39 (t, 2H), 7.16-7.30 (m, 6H), 6.66-6.69 (d, 2H), 3.94 (s, 3H); ^{13}C-NMR (75 MHz, $CDCl_3$): δ 160.4, 141.3, 133.0, 131.6, 131.4, 123.5, 122.7, 122.2, 117.6, 116.6, 55.9; MS (EI), m/z: [M^+], calcd. for $C_{19}H_{15}NO_3S$, 337.08; found, 336.98; analysis (calcd., found for $C_{19}H_{15}NO_3S$): C (67.64, 67.53), H (4.48, 4.22), N (4.15, 4.29), S (9.50, 9.75).

CS-CH_3: White solid (yield: 72%). mp: 231 °C; ^{1}H-NMR (300 MHz, $CDCl_3$): δ 8.15-8.18 (dd, 2H), 7.47-7.50 (d, 2H), 7.35-7.41 (m, 2H), 7.21-7.26 (m, 4H), 6.64-6.67 (d, 2H), 2.52 (s, 3H); ^{13}C-NMR (75 MHz, $CDCl_3$): δ 141.1, 140.1, 136.4, 132.9, 132.2, 130.3, 123.5, 122.7, 122.1, 117.5, 21.6; MS (EI), m/z: [M^+], calcd. for $C_{19}H_{15}NO_2S$, 321.08; found, 320.98; analysis (calcd., found for $C_{19}H_{15}NO_2S$): C (71.01, 71.28), H (4.70, 4.52), N (4.36, 4.67), S (9.98, 10.09).

CS-H: White solid (yield: 85%). mp: 206 °C; ^{1}H-NMR (300 MHz, $CDCl_3$): δ 8.16-8.19 (d, 2H), 7.63-7.73 (m, 3H), 7.36-7.40 (m, 4H), 7.22-7.26 (m, 2H), 6.61-6.63 (d, 2H); ^{13}C-NMR (75 MHz, $CDCl_3$): δ 140.7, 138.8, 132.7, 131.2, 130.3, 129.7, 123.3, 122.5, 121.9, 117.1; MS (EI), m/z: [M^+], calcd. for $C_{18}H_{13}NO_2S$, 307.07; found, 306.97; analysis (calcd., found for $C_{18}H_{13}NO_2S$): C (70.34, 70.36), H (4.26, 4.11), N (4.56, 4.69), S (10.43, 10.65).

CS-Br: White solid (yield: 55%). mp: 224 °C; ^{1}H-NMR (400 MHz, $CDCl_3$): δ 8.16-8.19 (m, 2H), 7.83-7.85 (m, 2H), 7.39-7.43 (m, 2H), 7.25-7.30 (m, 4H), 6.61-6.64 (m, 2H); ^{13}C-NMR (75 MHz, $CDCl_3$): δ 140.4, 137.8, 134.6, 132.8, 132.2, 123.8, 123.4, 122.8, 122.2, 116.9; MS (EI), m/z: [M^+], calcd. for $C_{18}H_{12}BrNO_2S$, 384.98; found, 384.83; analysis (calcd., found for $C_{18}H_{12}BrNO_2S$): C (55.97, 55.72), H (3.13, 3.41), N (3.63, 3.92), S (8.30, 8.11).

CS-Cl: White solid (yield: 45%). mp: 242 °C; ^{1}H-NMR (400 MHz, $CDCl_3$): δ 8.17-8.19 (m, 2H), 7.67-7.70 (m, 2H), 7.39-7.43 (m, 2H), 7.34-7.36 (m, 2H), 7.25-7.29 (m, 2H), 6.61-6.63 (m, 2H); ^{13}C-NMR (100 MHz, $CDCl_3$): δ 140.6, 137.3, 135.9, 132.9, 132.0, 131.7, 123.5, 122.8, 122.3, 117.0; MS (EI), m/z: [M^+], calcd. for $C_{18}H_{12}ClNO_2S$, 341.03; found, 340.92; analysis (calcd., found for $C_{18}H_{12}ClNO_2S$): C (63.25, 63.30), H (3.54, 3.82), N (4.10, 4.20), S (9.38, 9.28).

CS-F: White solid (yield: 67%). mp: 247 °C; ^{1}H-NMR (400 MHz, $CDCl_3$): δ 8.16-8.18 (m, 2H), 7.38-7.43 (m, 6H), 7.24-7.28 (m, 2H), 6.61-6.63 (m, 2H); ^{13}C-NMR (100 MHz, $CDCl_3$): δ 164.0, 161.5, 140.7, 134.6, 132.8, 132.4, 132.3, 123.4, 122.7, 122.2, 118.5, 118.3, 117.0; MS (EI), m/z: [M^+], calcd. for $C_{18}H_{12}FNO_2S$, 325.06; found, 324.95; analysis (calcd., found for $C_{18}H_{12}FNO_2S$): C (66.45, 66.22), H (3.72, 3.50), N (4.31, 4.50), S (9.85, 10.07).

CS-CF_3: White solid (yield: 72%). mp: 252 °C; ^{1}H-NMR (400 MHz, $CDCl_3$): δ 8.00-8.03 (t, 4H), 7.58-7.61 (d, 2H), 7.40-7.45 (t, 2H), 7.24-7.29 (t, 2H), 6.62-6.65 (d, 2H); ^{13}C-NMR (100 MHz, $CDCl_3$): δ 142.14, 142.13, 140.31, 133.01, 132.22, 131.89, 131.43, 128.67, 128.63, 128.60, 128.56, 124.88, 123.64, 122.91, 122.54, 122.17, 116.88; MS (EI), m/z: [M^+], calcd. for $C_{19}H_{12}F_3NO_2S$, 375.05; found, 374.94; analysis (calcd., found for $C_{19}H_{12}F_3NO_2S$): C (60.80, 61.04), H (3.22, 3.24), N (3.73, 3.92), S (8.54, 8.34).

Supplementary Figures

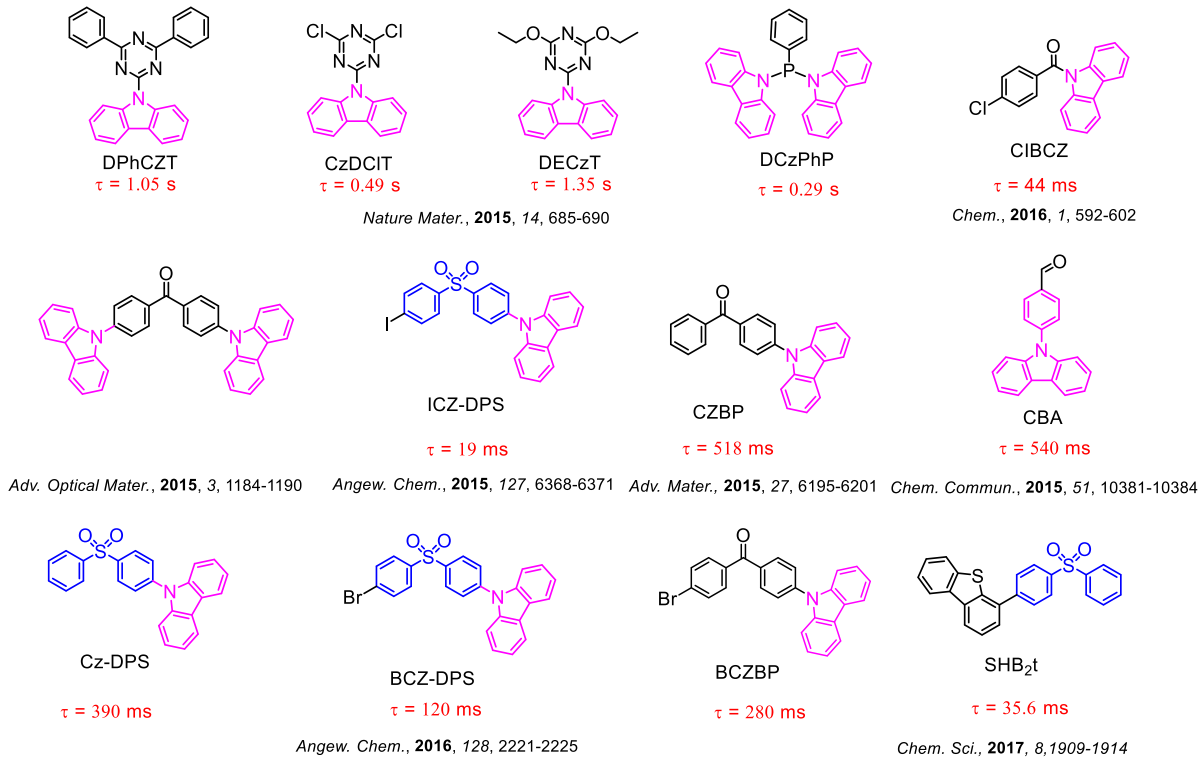

Supplementary Figure 1 Molecular structures and the corresponding lifetimes of the reported persistent RTP materials containing the groups of sulfonyldibenzene or carbazole.[1-8]

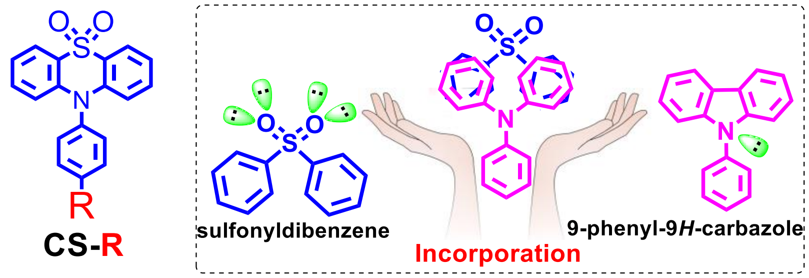

Supplementary Figure 2 The molecular design strategy for purely organic persistent room temperature phosphorescence (RTP) materials: the introduction of sulfonyldibenzene and 9-phenyl-9*H*-carbazole, of which the existence of O or N atoms are capable of promoting *n*–*π** transitions to populate triplet excitons, and would be much beneficial to the RTP character.

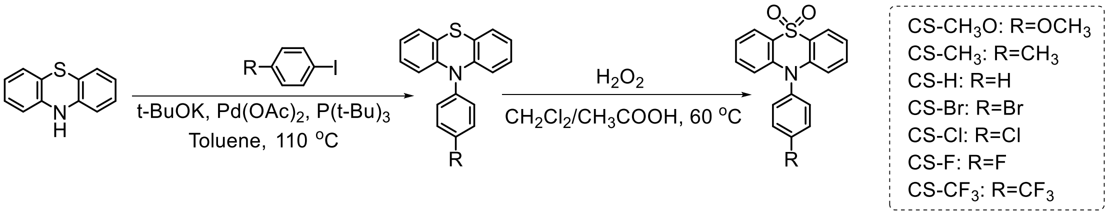

Supplementary Figure 3 The synthetic route of **CS-CH$_3$O, CS-CH$_3$, CS-H, CS-Br, CS-Cl, CS-F** and **CS-CF$_3$**.

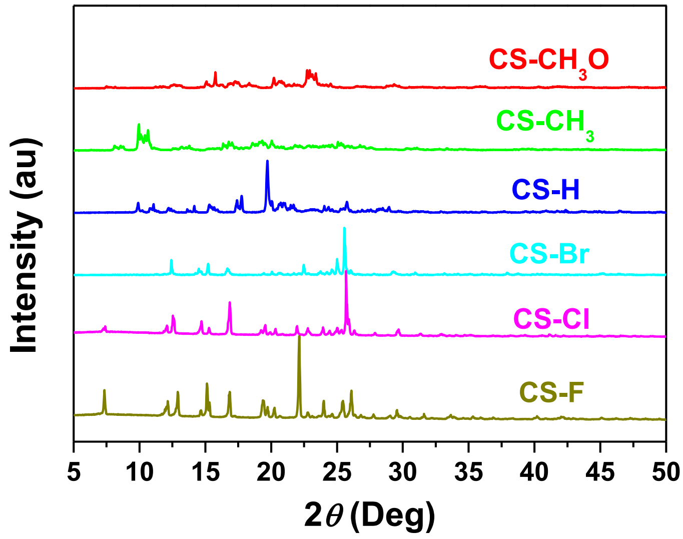

Supplementary Figure 4 The PXRD patterns for the as-prepared powders of **CS-CH₃O**, **CS-CH₃, CS-H**, **CS-Br**, **CS-Cl** and **CS-F**.

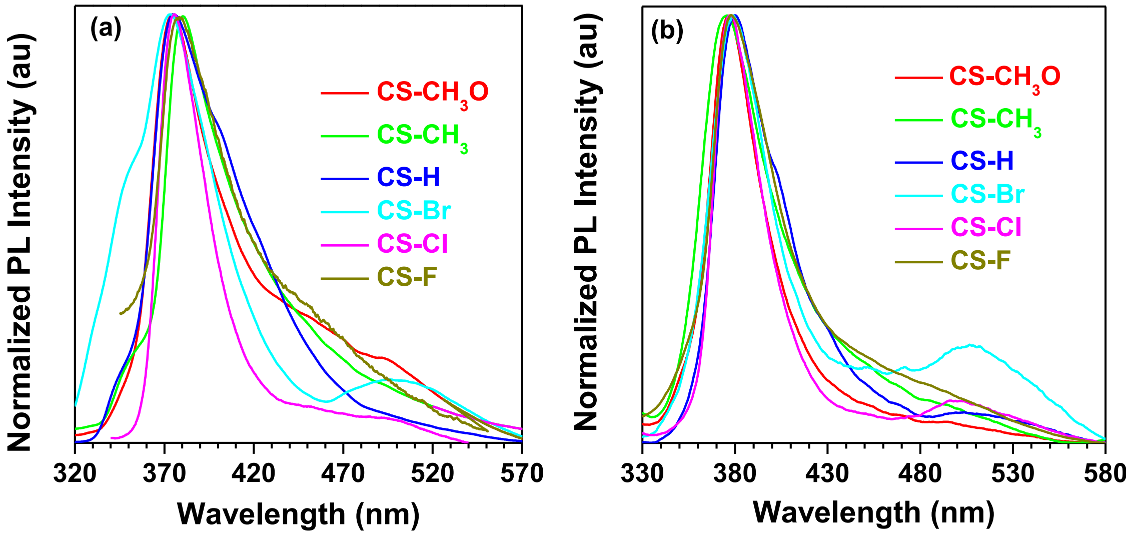

Supplementary Figure 5 The normalized fluorescence spectra of as-prepared powders **(a)** and crystals **(b)** of **CS-CH₃O**, **CS-CH₃**, **CS-H**, **CS-Br**, **CS-Cl** and **CS-F**.

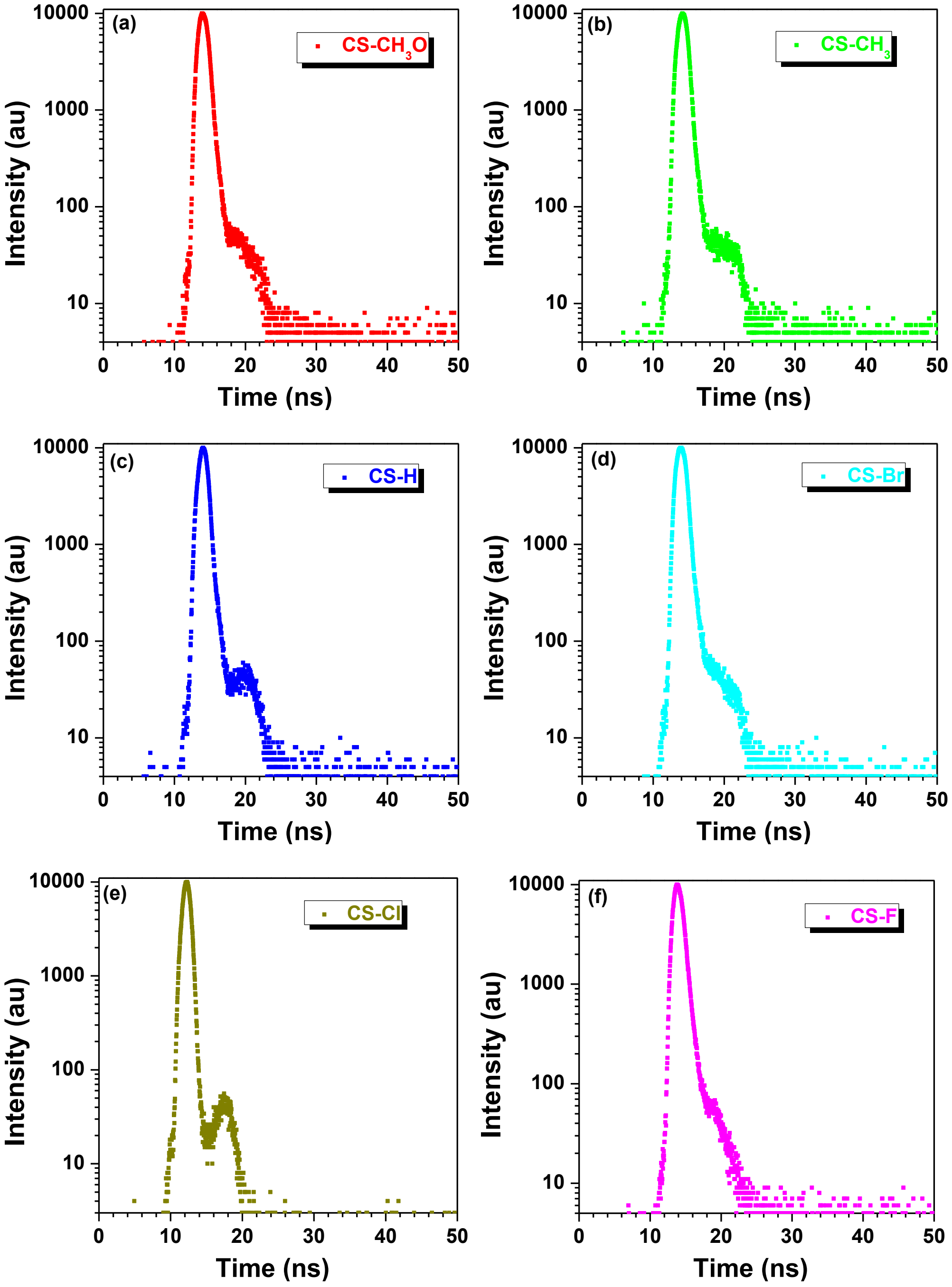

Supplementary Figure 6 Fluorescence decay of as-prepared powders of **CS-CH_3O (a)**, **CS-CH_3 (b)**, **CS-H (c)**, **CS-Br (d)**, **CS-Cl (e)** and **CS-F (f)**.

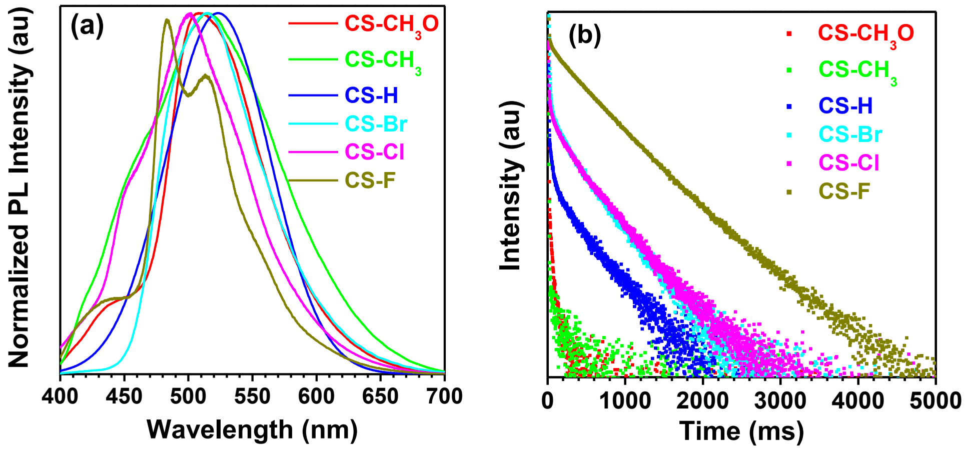

Supplementary Figure 7 The normalized phosphorescence spectra **(a)** and the corresponding lifetimes **(b)** of as-prepared powders for **CS-CH₃O, CS-CH₃, CS-H, CS-Br, CS-Cl** and **CS-F**.

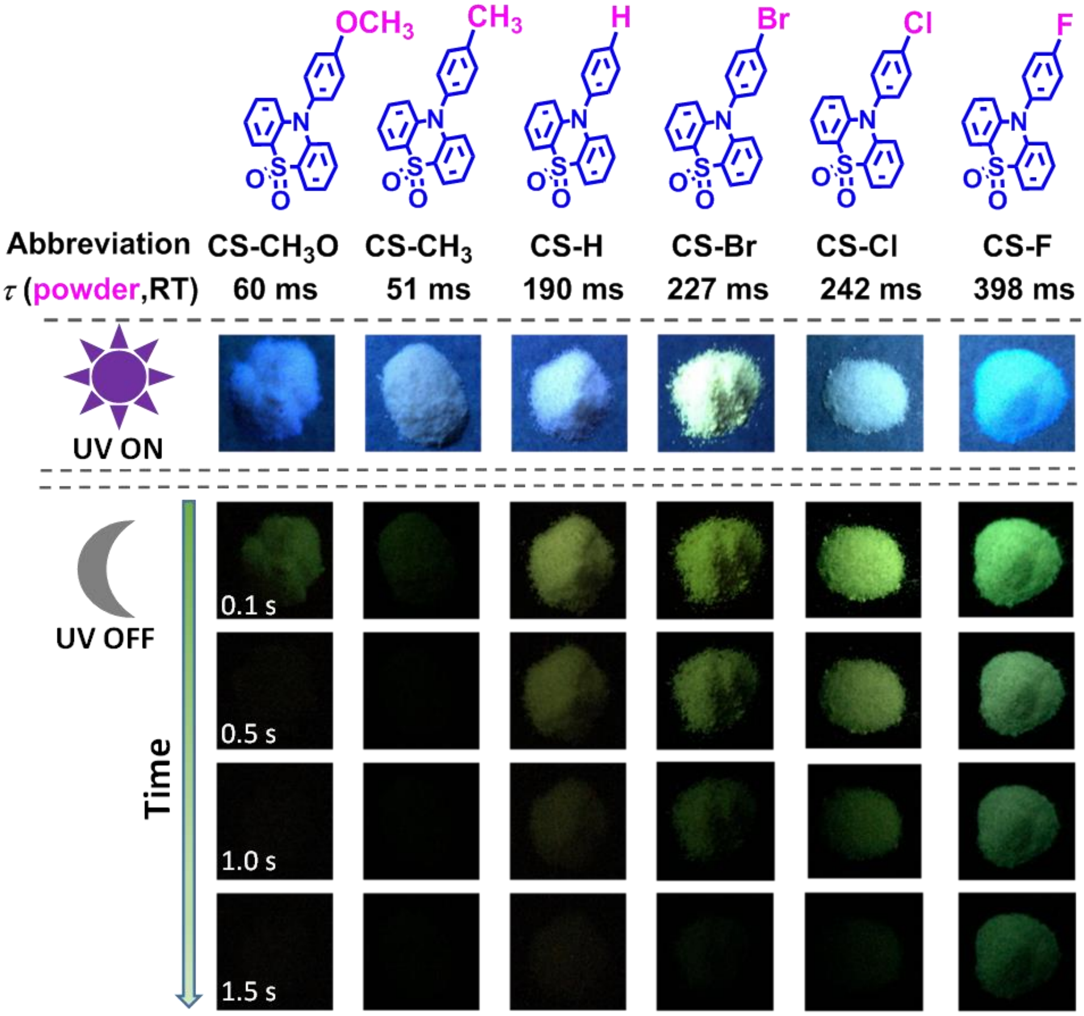

Supplementary Figure 8 The molecular structures of the six room temperature phosphorescence (RTP) compounds of **CS-CH₃O**, **CS-CH₃**, **CS-H**, **CS-Br**, **CS-Cl**, and **CS-F**, and their corresponding phosphorescence lifetimes in powders at room temperature (RT). The photographs were taken at different time, before and after turning-off the 365 nm UV-irritation under ambient conditions.

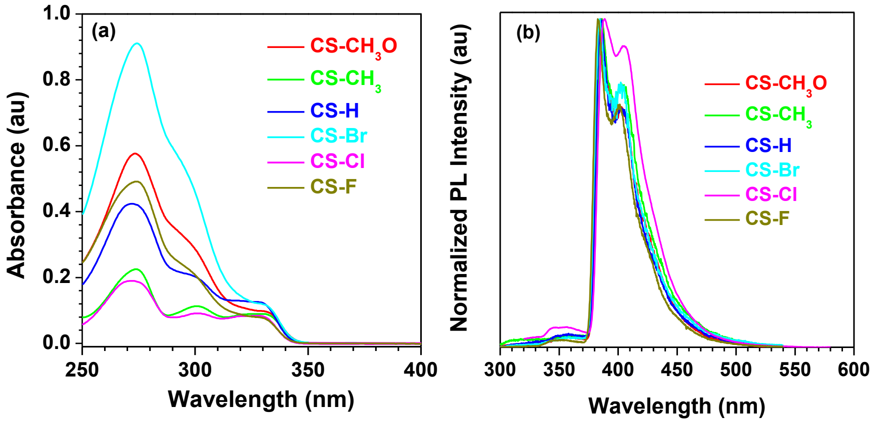

Supplementary Figure 9 (a) The UV-visible spectra of the dilute dichloromethane solutions of **CS-CH₃O**, **CS-CH₃**, **CS-H**, **CS-Br**, **CS-Cl** and **CS-F** at room temperature (Concentration: 10^{-5} M); **(b)** The PL spectra of the dilute dichloromethane solutions of **CS-CH₃O**, **CS-CH₃**, **CS-H**, **CS-Br**, **CS-Cl** and **CS-F** at 77K (Concentration: 10^{-5} M)

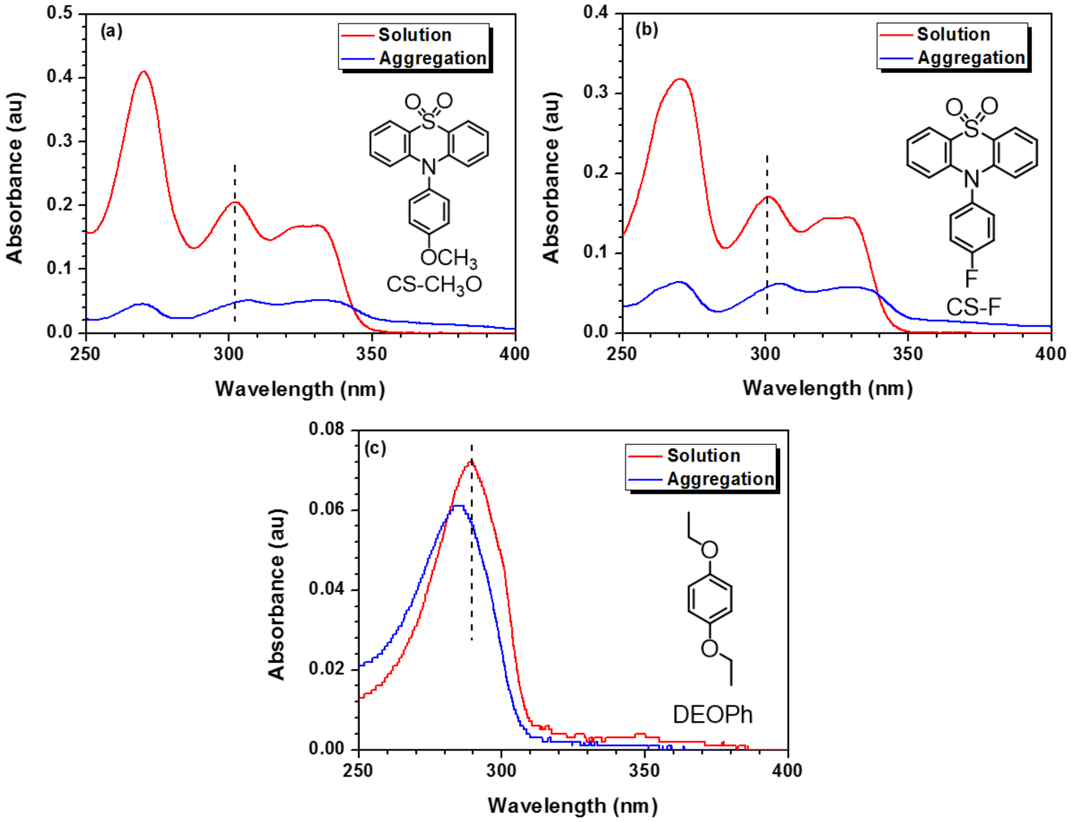

Supplementary Figure 10 (a) The UV-visible spectra of **CS-CH₃O** in methanol solution and aggregation (methanol/water mixtures with f_{water} = 99%); **(b)** The UV-visible spectra of **CS-F** in methanol solution and aggregation state (methanol/water mixtures with f_{water} = 99%); **(c)** The UV-visible spectra of **DEOPh** in methanol solution and aggregation state (methanol/water mixtures with f_{water} = 99%).

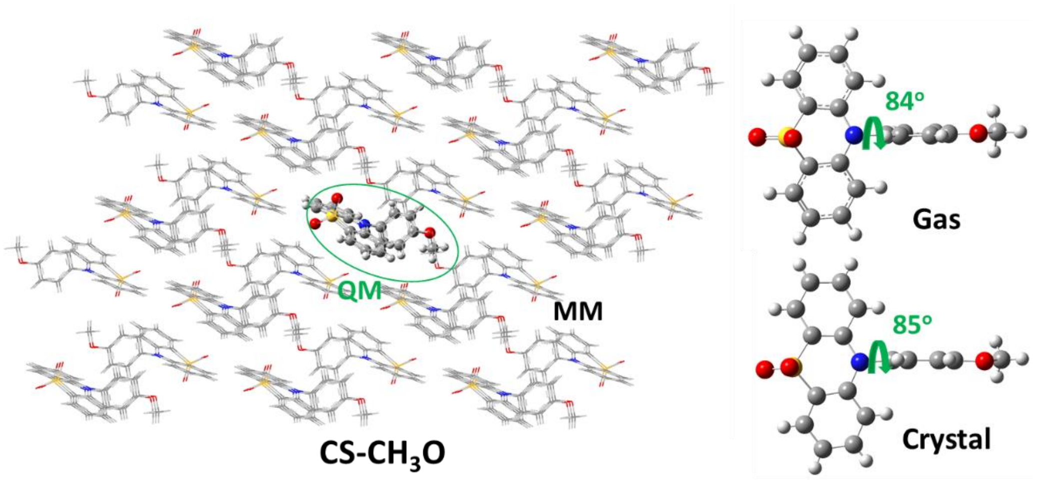

Supplementary Figure 11 The QM/MM model for **CS-CH₃O**: one central QM molecule for high layer and the surrounding 62 MM molecules for low layer. The optimized molecular structure in gas state and the selected molecular structure in crystal for **CS-CH₃O**.

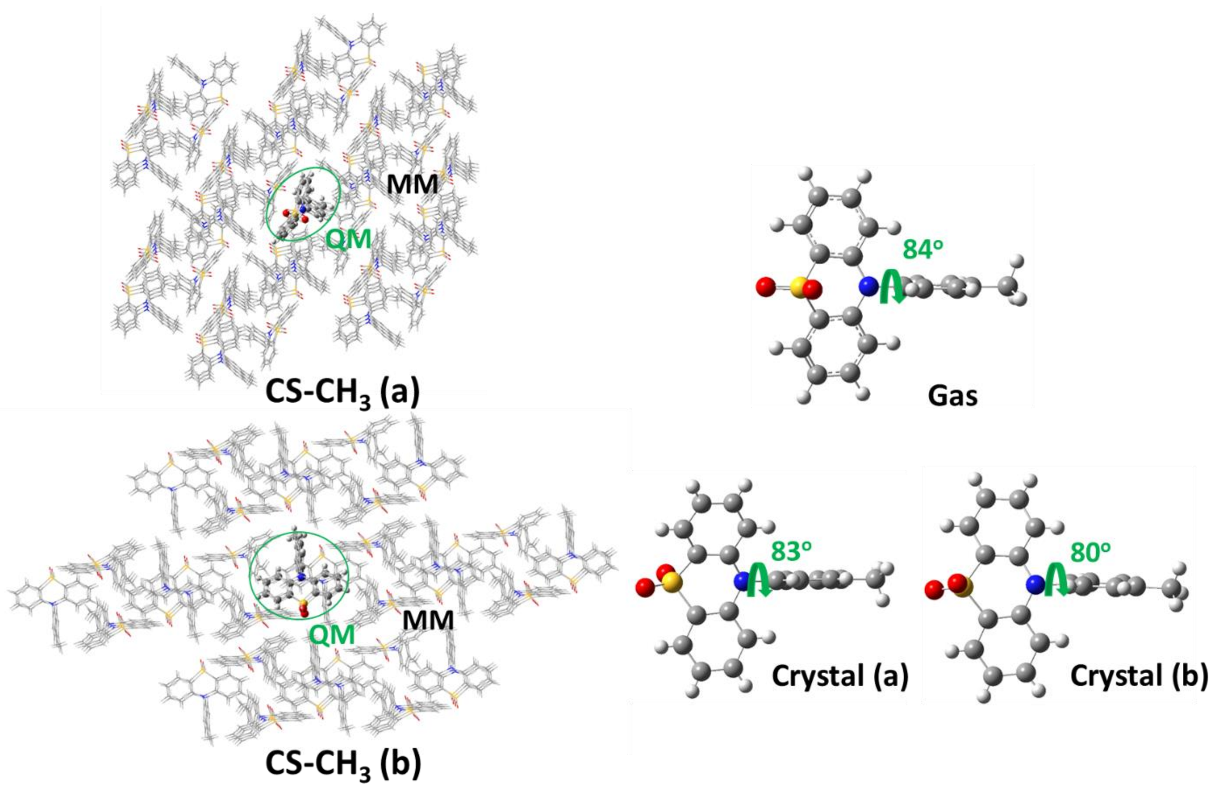

Supplementary Figure 12 The QM/MM model for **CS-CH₃**: one central QM molecule for high layer and the surrounding 88 MM molecules for low layer. The optimized molecular structure in gas state and the selected molecular structures in crystal for **CS-CH₃**.

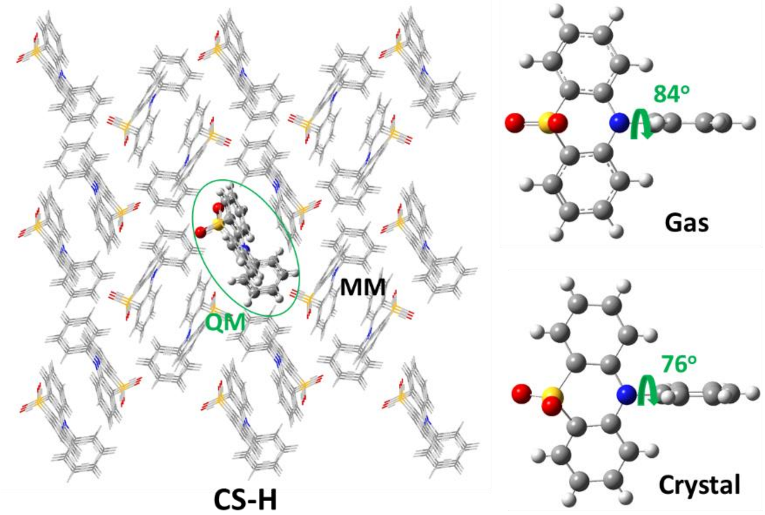

Supplementary Figure 13 The QM/MM model for **CS-H**: one central QM molecule for high layer and the surrounding 62 MM molecules for low layer. The optimized molecular structure in gas state and the selected molecular structure in crystal for **CS-H**.

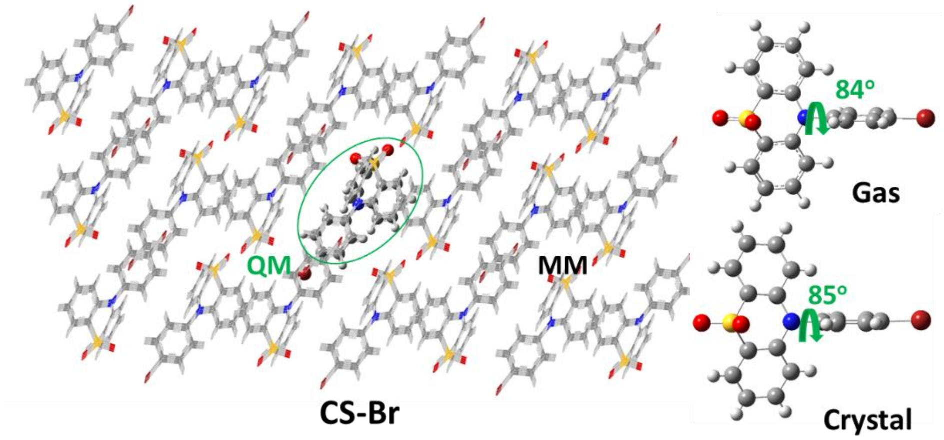

Supplementary Figure 14 The QM/MM model for **CS-Br**: one central QM molecule for high layer and the surrounding 104 MM molecules for low layer. The optimized molecular structure in gas state and the selected molecular structure in crystal for **CS-Br**.

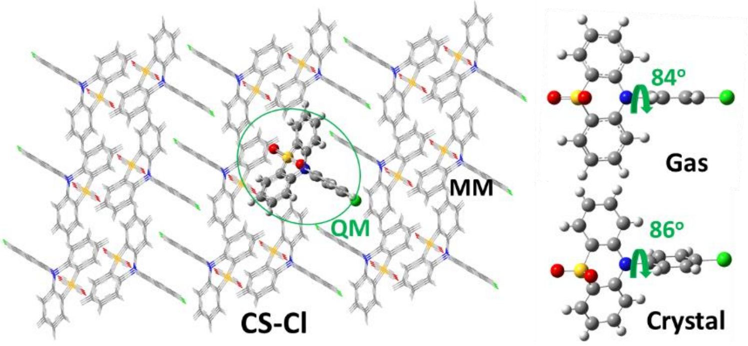

Supplementary Figure 15 The QM/MM model for **CS-Cl**: one central QM molecule for high layer and the surrounding 53 MM molecules for low layer. The optimized molecular structure in gas state and the selected molecular structure in crystal for **CS-Cl**.

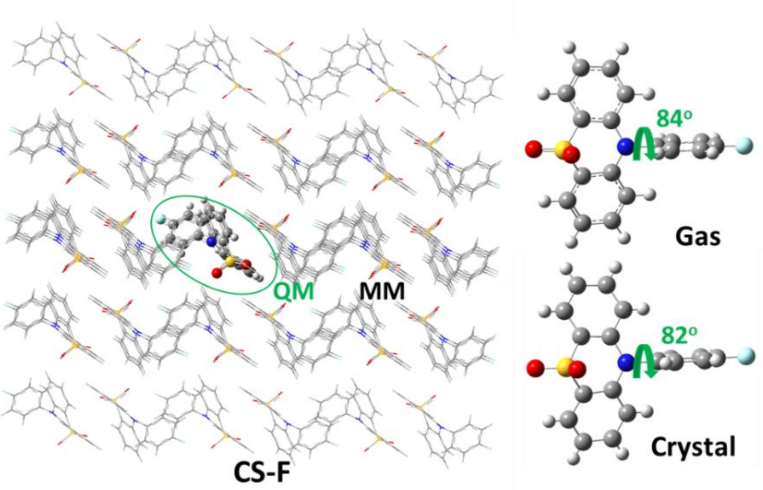

Supplementary Figure 16 The QM/MM model for **CS-F**: one central QM molecule for high layer and the surrounding 53 MM molecules for low layer. The optimized molecular structure in gas state and the selected molecular structure in crystal for **CS-F**.

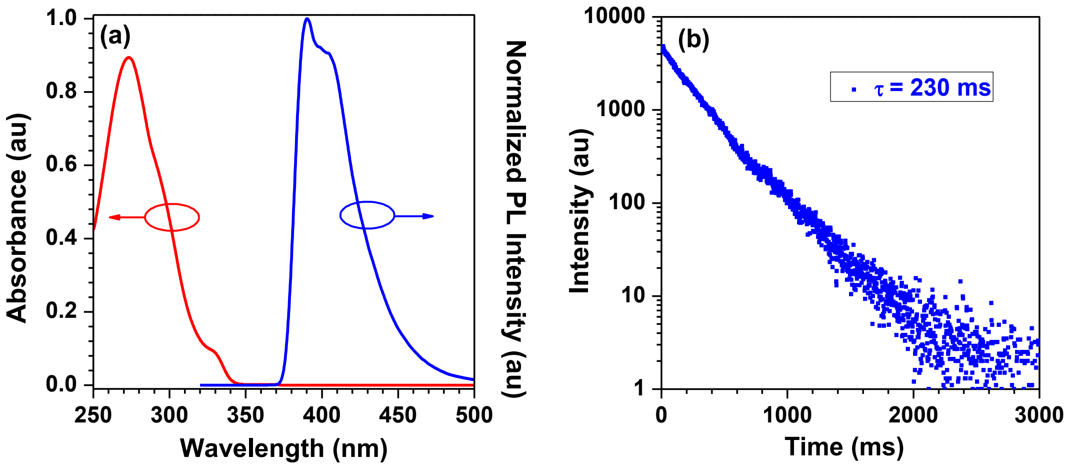

Supplementary Figure 17 (a) The UV-visible spectrum and the normalized low temperature (77K) phosphorescence spectrum of **CS-CF$_3$** in dilute dichloromethane solution; **(b)** The time-resolved PL-decay curves for their low temperature (77K) phosphorescence in dilute dichloromethane solution, concentration: 10^{-5} M.

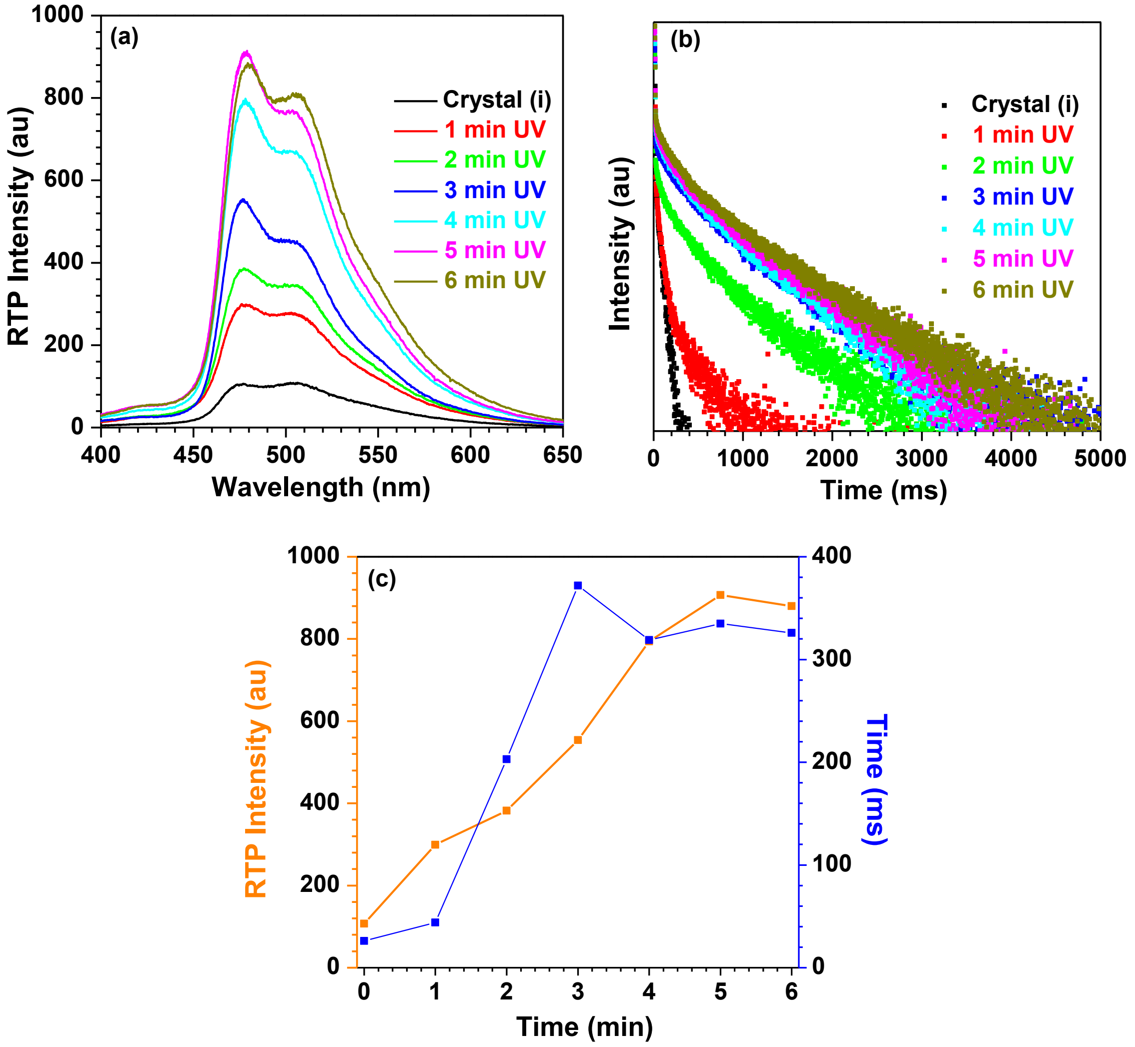

Supplementary Figure 18 (a) RTP spectra of **CS-CF_3** crystal with increasing irradiation time from 1 to 6 min; **(b)** Time-resolved PL-decay curves for the room temperature phosphorescence of **CS-CF_3** crystal with increasing irradiation time from 1 to 6 min; **(c)** Variation tendency for RTP intensity and lifetime of **CS-CF_3** crystal with increasing irradiation time from 1 to 6 min.

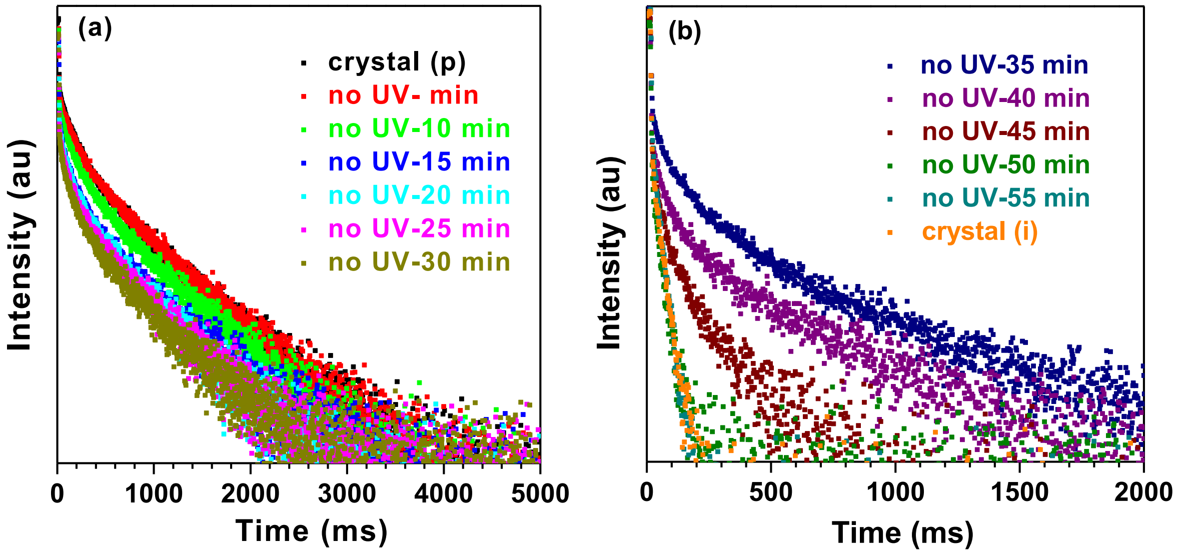

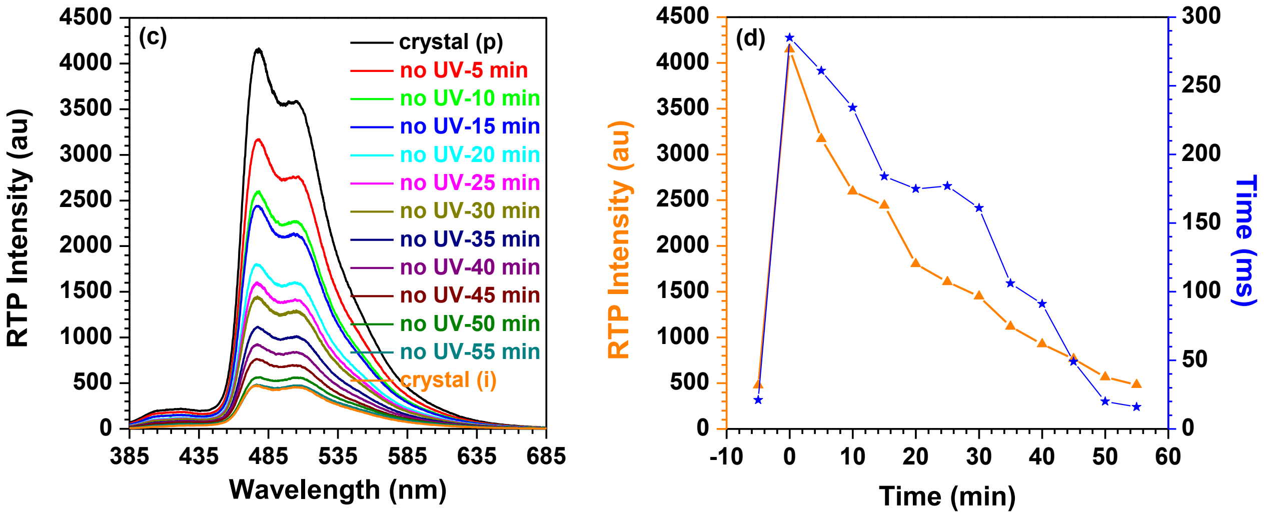

Supplementary Figure 19 (a) Time-resolved PL-decay curves for the room temperature phosphorescence of **CS-CF$_3$** crystal with increasing standing time from 5 to 30 min; **(b)** Time-resolved PL-decay curves for the room temperature phosphorescence of **CS-CF$_3$** crystal with increasing standing time from 35 to 55 min; **(c)** RTP spectra of **CS-CF$_3$** crystal with increasing standing time from 5 to 55 min; **(d)** Variation tendency for RTP intensity and lifetime of **CS-CF$_3$** crystal in different time: initial state to phosphorescence state, then to standing different time.

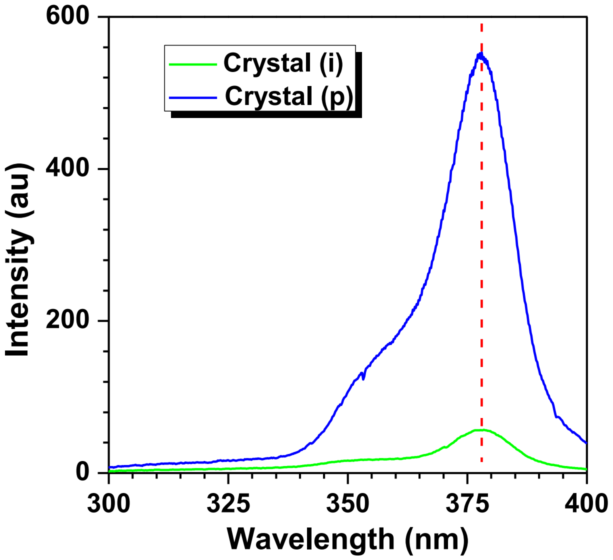

Supplementary Figure 20 The phosphorescence excitation spectra (@480 nm) of **CS-CF$_3$** crystal before and after five minutes 365 nm UV-irradiation.

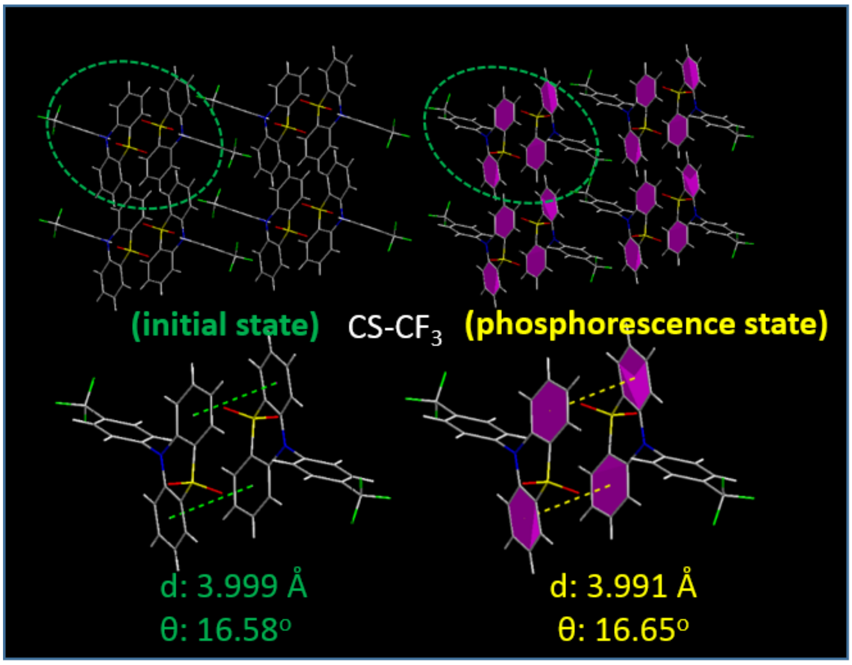

Supplementary Figure 21 Global and local packing of the crystals for initial and phosphorescence state **CS-CF₃**: the local packing pictures were selected from the parts in cycles of corresponding global ones, in which the centroid–centroid distances (d) and the dihedral angles (θ) of the involved phenyl rings are listed and the phenyl rings involving in the stronger *π*-*π* interactions are labeled by pink polyhedrons.

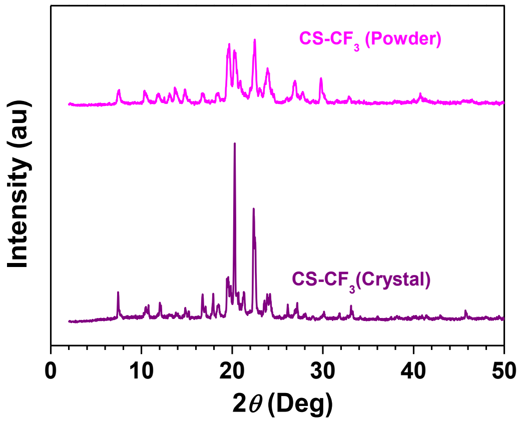

Supplementary Figure 22 The PXRD patterns for the as-prepared powder and crystal of **CS-CF₃** in which the as-prepared **CS-CF₃** displays low-intensity reflections indicating the low degree of crystallization.

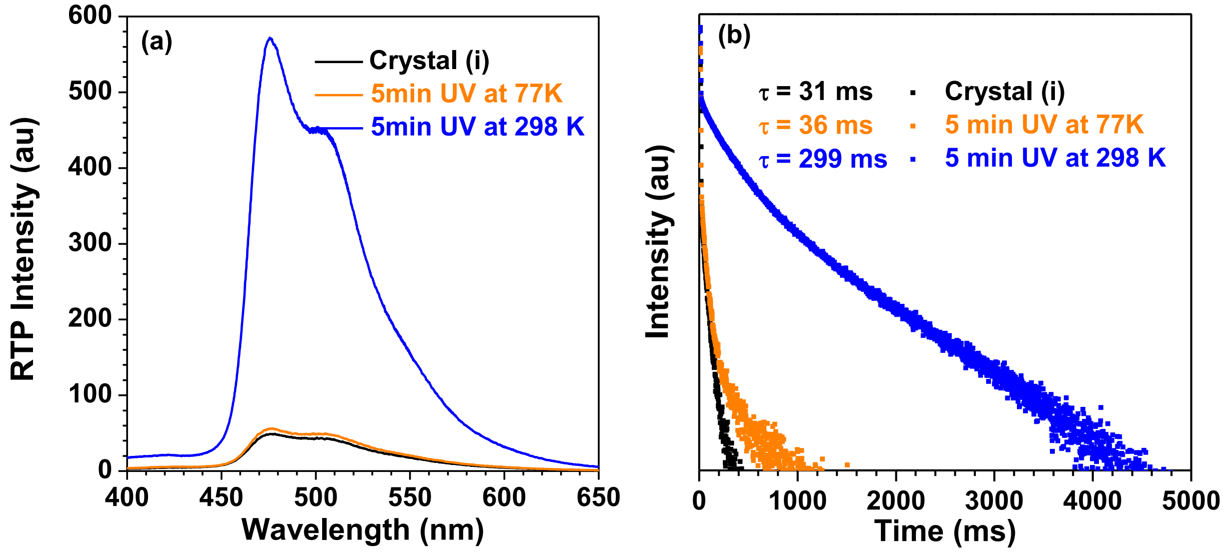

Supplementary Figure 23 (a) RTP spectra of **$CS\text{-}CF_3$** crystal at initial state and after five minutes 365 nm UV-irradiation at 77 K or 298 K; **(b)** Time-resolved PL-decay curves for the room temperature phosphorescence of **$CS\text{-}CF_3$** crystal at initial state and after five minutes 365 nm UV-irradiation at 77 K or 298 K.

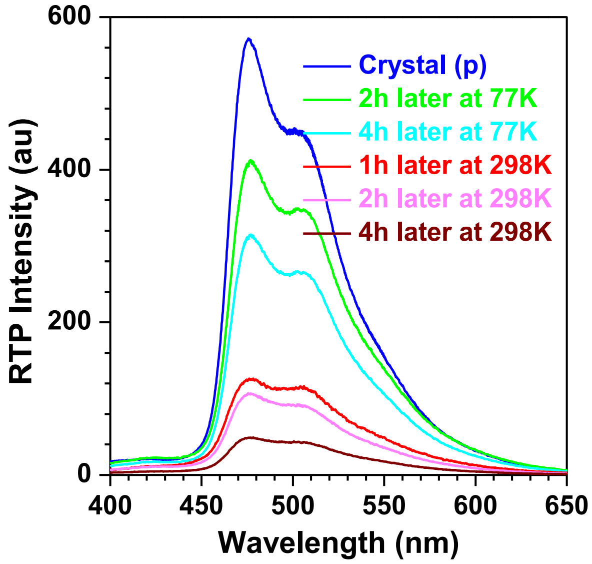

Supplementary Figure 24 Room temperature phosphorescence intensity of **$CS\text{-}CF_3$** crystal under five minutes 365 nm UV-irradiation and after one or two or four hours standing at 77 K or 298 K.

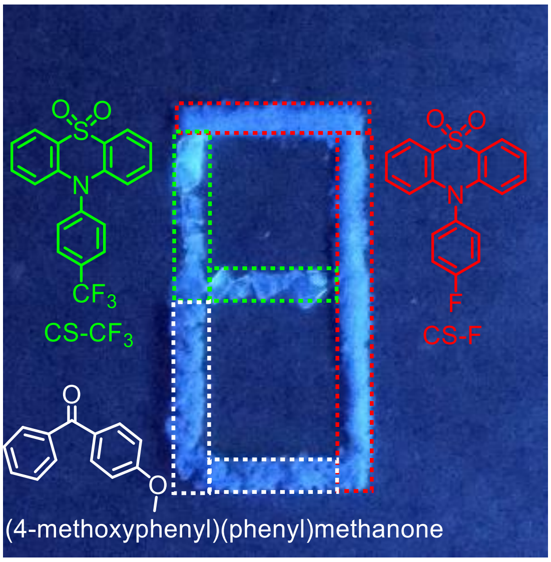

Supplementary Figure 25 The components of the pattern '8'8: one is **CS-CF$_3$** with reversible photo-induced phosphorescence change character (in green dashed box), another is **CS-F** with normally persistent RTP character (in red dashed box), last is (4-methoxyphenyl)(phenyl)methanone without RTP effect (in white dashed box).

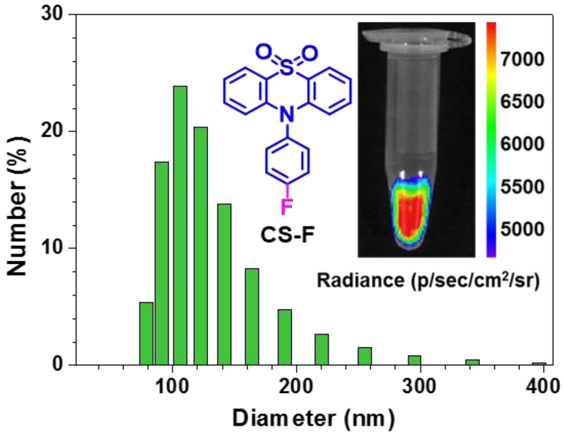

Supplementary Figure 26 The size distribution of organic nanoparticles based on **CS-F** (Inset: Ultralong phosphorescence images of **CS-F** nanoparticles solution, taken at t =10 s after removal of light illumination source).

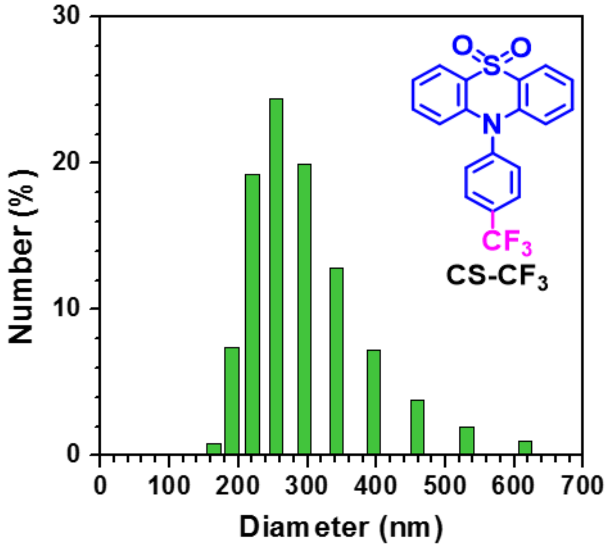

Supplementary Figure 27 The size distribution of organic nanoparticles based on **CS-CF$_3$.**

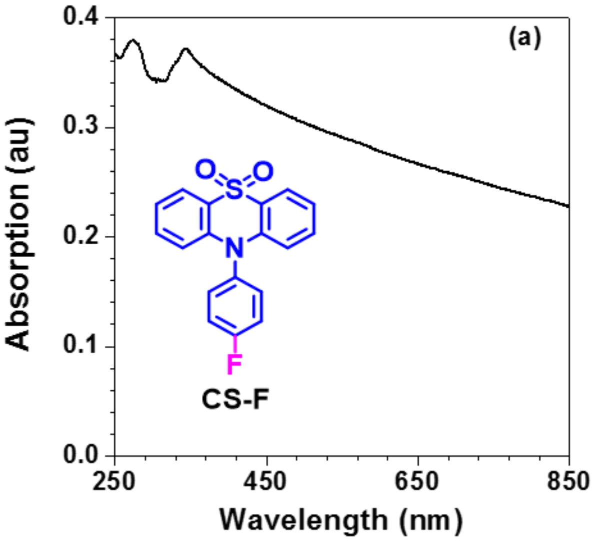

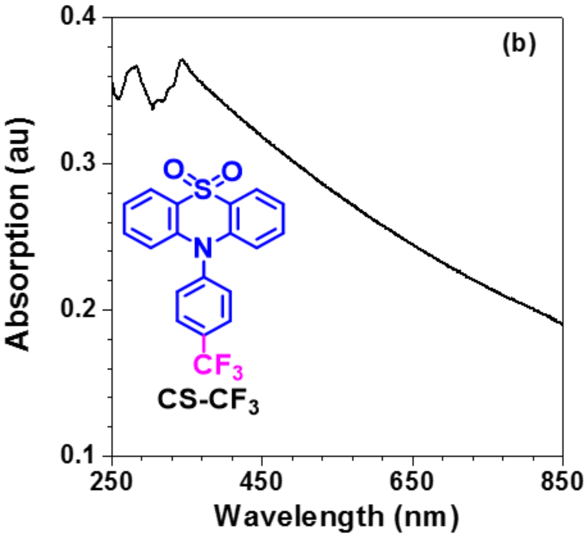

Supplementary Figure 28 (a) The UV-visible spectrum of the **CS-F** nanoparticles; **(b)** The UV-visible spectrum of the **CS-CF$_3$** nanoparticles.

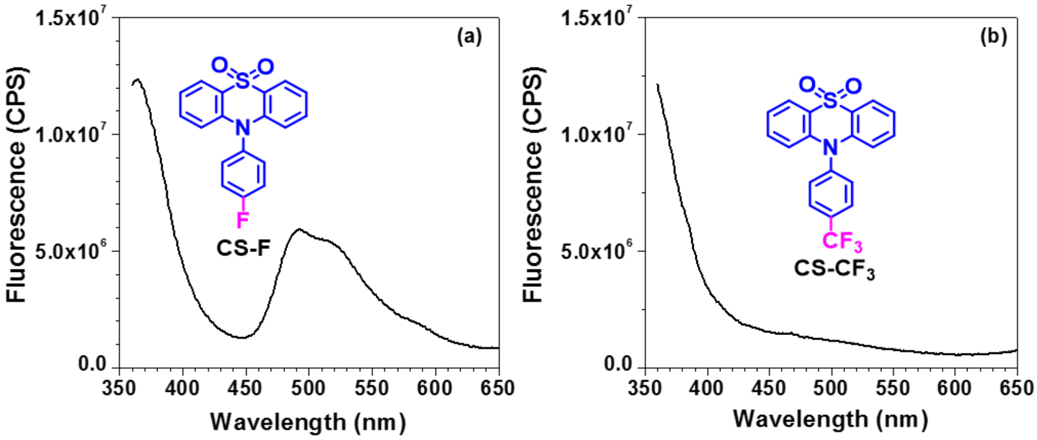

Supplementary Figure 29 (a) Prompt emission spectrum of **CS-F** nanoparticles; **(b)** Prompt emission spectrum of **$CS-CF_3$** nanoparticles.

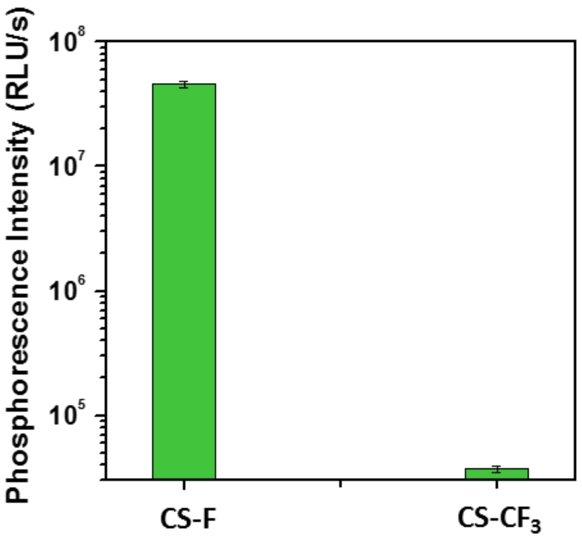

Supplementary Figure 30 The ultralong phosphorescence intensities, measured at t =10 s after removal of light illumination source, of **CS-F** and **$CS-CF_3$** nanoparticles.

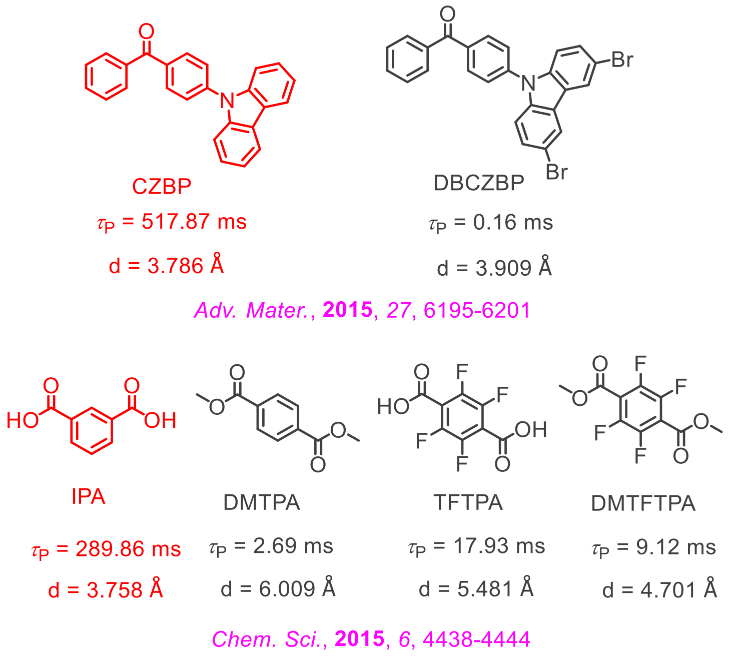

Supplementary Figure 31 The molecular structures, corresponding lifetimes and the π-π distances of the involved aromatic rings in single crystals for some reported RTP materials, in which the molecules with strong π-π interactions show persistent RTP effect while others with weak π-π interactions give much short RTP lifetimes.[5,9]

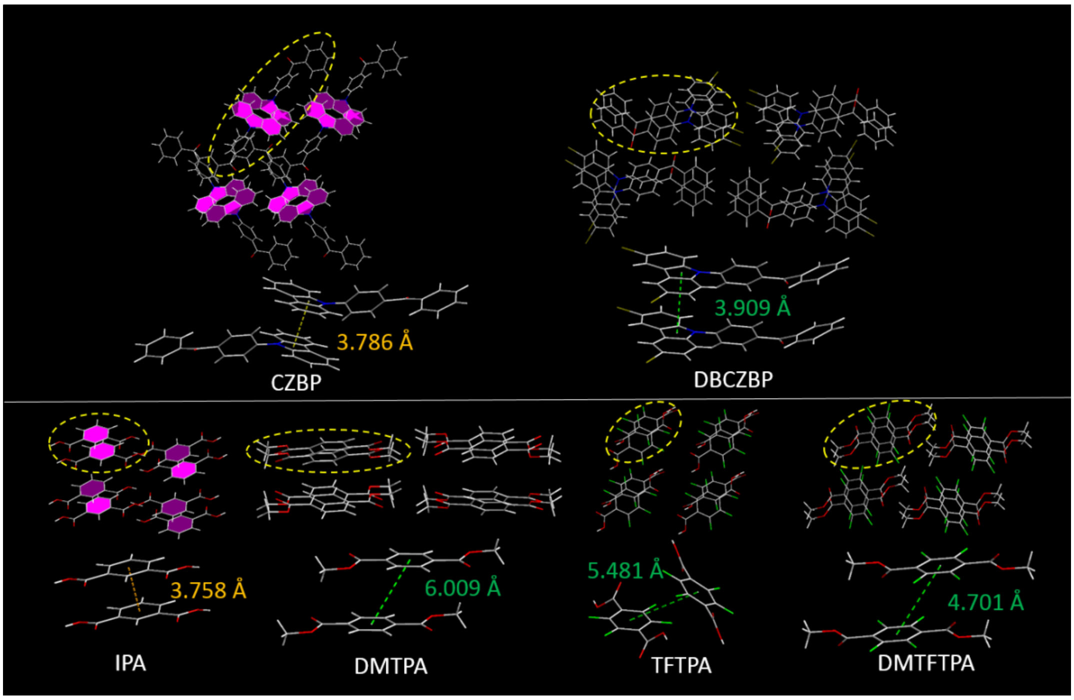

Supplementary Figure 32 Global and local packing modes of the crystals for **CZBP**, **DBCZBP**, **IPA**, **DMTPA**, **TFTPA**, **DMTFTPA**: the local packing pictures were selected from the parts in cycles of corresponding global ones, in which the centroid–centroid distances of the involved aromatic rings are listed and the phenyl rings involved in the stronger π-π interactions in global are labeled by pink color.

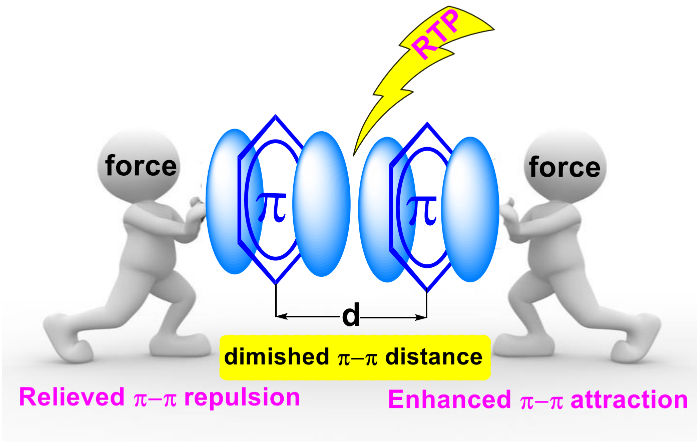

Supplementary Figure 33 A proposed diagram to enhance the π-π interaction and promote the persistent RTP**:** The strong π-π interactions in solid could stabilize the T_1 state and lead to the persistent RTP. There are mainly two driving forces to diminish the π-π distance, one is the relieved π-π repulsion (decreasing the π-electron density or reducing the steric hindrance *et al.*) and another is the enhanced π-π attraction (electrostatic interactions or dipole-dipole interactions *et al.*).

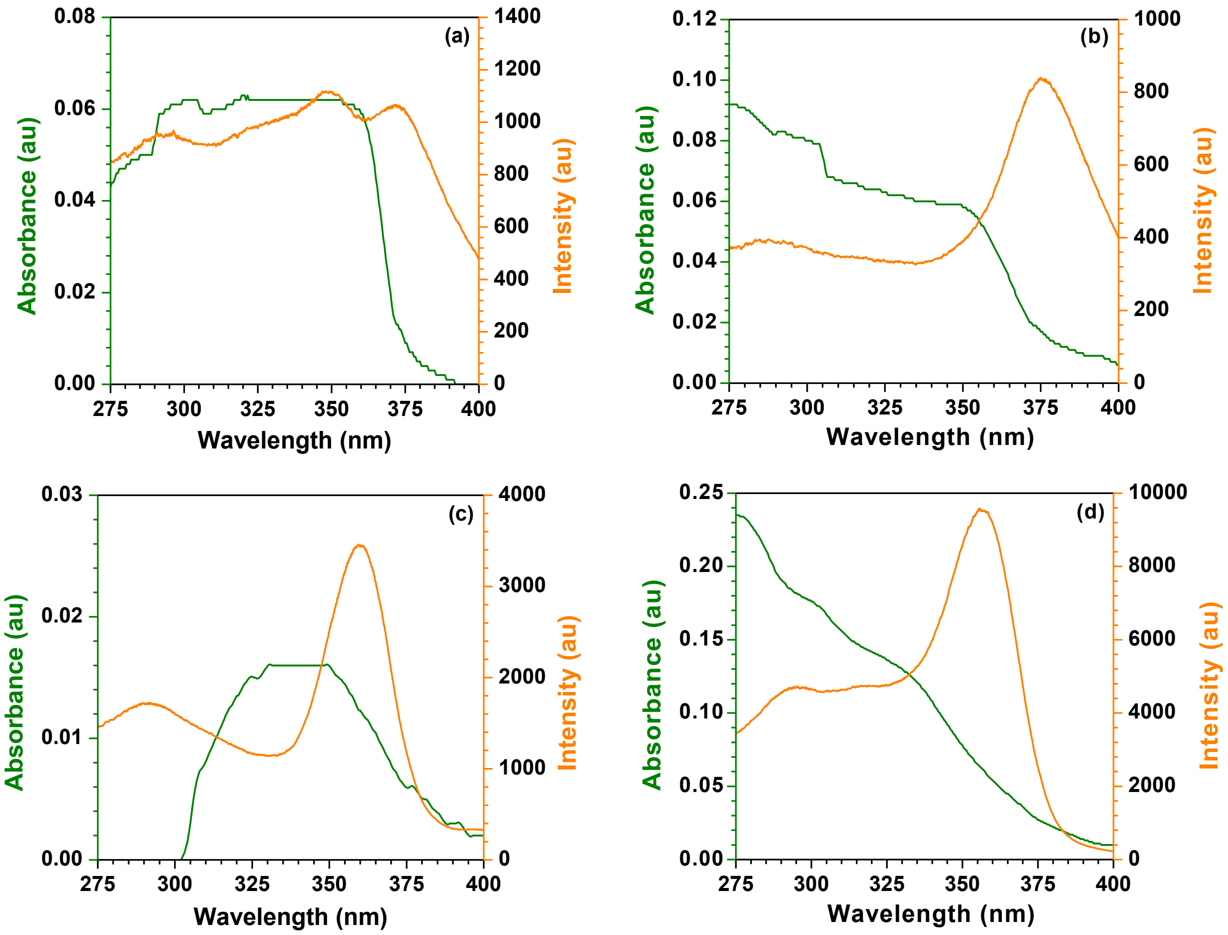

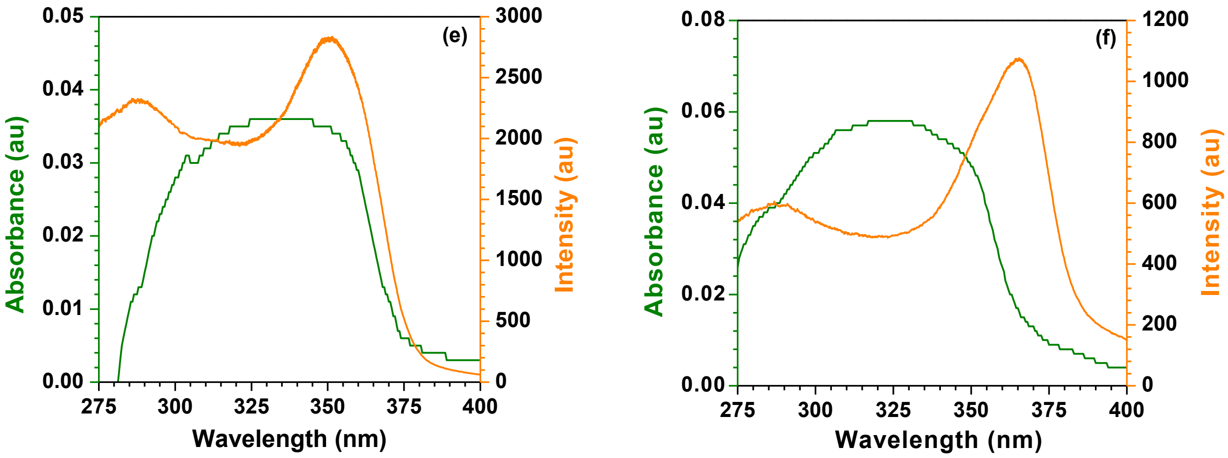

Supplementary Figure 34 (a) The phosphorescence excitation spectrum (@ 510 nm) and absorption spectrum of **CS-CH₃O** crystal; **(b)** The phosphorescence excitation spectrum (@ 515 nm) and absorption spectrum of **CS-CH₃** crystal; **(c)** The phosphorescence excitation spectrum (@ 515 nm) and absorption spectrum of **CS-H** crystal; **(d)** The phosphorescence excitation spectrum (@ 515 nm) and absorption spectrum of **CS-Br** crystal; **(e)** The phosphorescence excitation spectrum (@ 500 nm) and absorption spectrum of **CS-Cl** crystal; **(f)** The phosphorescence excitation spectrum (@ 485 nm) and absorption spectrum of **CS-F** crystal.

Supplementary Tables

Supplementary Table 1 The photophysical data of **CS-CH₃O**, **CS-CH₃**, **CS-H**, **CS-Br**, **CS-Cl** and **CS-F** in solutions and crystals.

Compound	$\lambda_{abs}{}^{a}$ (nm)	$\lambda_{F}{}^{b}$ (nm)	$\tau_{F}{}^{b}$ (ns)	$\lambda_{P}{}^{c}$ (nm)	$\tau_{P}{}^{c}$ (ms)	$\lambda_{P}{}^{d}$ (nm)	$\tau_{P}{}^{d}$ (ms)	Φ_{PL} (%)
CS-CH3O	331	375	0.35	509	88	389, 405	242	3.07
CS-CH3	331	380	0.34	516	96	389,402	268	4.21
CS-H	329	375	0.29	525	188	386,404	225	3.11
CS-Br	329	373	0.38	525	268	389,405	248	3.59
CS-Cl	329	376	0.66	500	256	389,402	240	10.16
CS-F	330	378	0.40	483, 513	410	389,403	222	5.52

[a] Observed from absorption spectra in dilute dichloromethane solution. [b] Determined in powder at room temperature. [c] Determined in crystal at room temperature. [d] Determined in solution at 77K.

Supplementary Table 2 Structure data of single crystals of **CS-CH_3O**, **CS-CH_3, CS-H**, **CS-Br**, **CS-C**l and **CS-F**.

Name	CS-CH_3O	CS-CH_3	CS-H	CS-Br	CS-Cl	CS-F
Formula	$C_{19}H_{15}NO_3S$	$C_{38}H_{30}N_2O_4S_2$	$C_{18}H_{13}NO_2S$	$C_{18}H_{12}BrNO_2S$	$C_{18}H_{12}ClNO_2S$	$C_{18}H_{12}FNO_2S$
Wavelength (Å)	1.54184	1.54184	1.54184	1.54184	0.71073	0.71073
Space Group	P21/n	P21/n	P21/n	P-1	P-1	P-1
Cell Lengths (Å)	a=13.0729(2)<br>b=8.67283(12)<br>c=15.1083(3)	a=16.8938(2)<br>b=10.39697(14)<br>c=18.1046(2)	a=8.91731(15)<br>b=12.8641(2)<br>c=12.7838(2)	a=8.1530(4)<br>b=8.6788(4)<br>c=12.2986(3)	a=8.1826(10)<br>b=8.6334(10)<br>c=12.3856(15)	a=8.0945(12)<br>b=8.3587(13)<br>c=12.4238(19)
Cell Angles (o)	α=90<br>β=111.697(2)<br>γ=90	α=90<br>β=104.5404(13)<br>γ=90	α=90.00<br>β=104.5577(17)<br>γ=90.00	α=96.241(3)<br>β=97.379(3)<br>γ=113.854(4)	α=96.744(2)<br>β=97.231(2)<br>γ=113.891(2)	α=98.324(3)<br>β=96.228(2)<br>γ=113.477(2)
Cell Volume (Å^3)	1591.60(5)	3078.11(7)	1419.39 (4)	776.83(5)	779.83(16)	750.0(2)
Z	4	4	4	2	2	2
Density (g/cm^3)	1.408	1.387	1.438	1.651	1.456	1.441
F(000)	704.0	1344.0	640.0	388.0	352.0	336.0
h_{max}, k_{max}, l_{max}	15, 10, 17	20, 12, 21	11, 15, 15	9, 10, 14	10,10,15	10, 10, 15
CCDC Number	1519854	1519853	1519540	1503919	1519837	1519635

Supplementary Table 3 The molecular conformations and HOMO, LUMO orbits for the isolated molecules and coupled units of **CS-CH$_3$O**, **CS-CH$_3$**, **CS-H**, **CS-Br**, **CS-Cl**, **CS-F** and **CS-CF$_3$**.

Single			Coupled units		
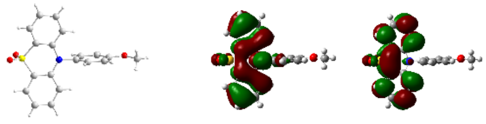			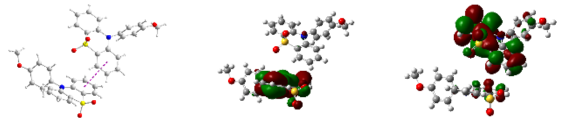		
CS-CH$_3$O	HOMO	LUMO	CS-CH$_3$O	HOMO	LUMO
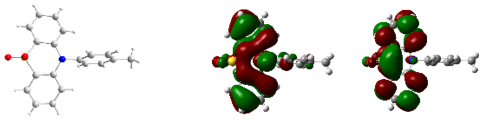			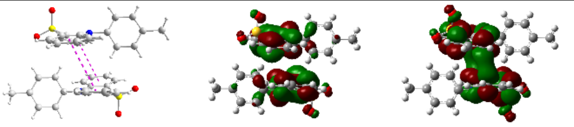		
CS-CH$_3$ (a)	HOMO	LUMO	CS-CH$_3$ (a)	HOMO	LUMO
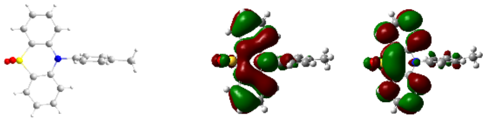			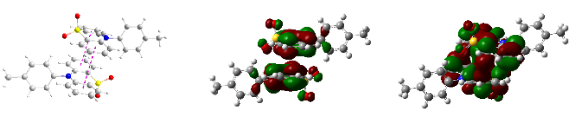		
CS-CH$_3$ (b)	HOMO	LUMO	CS-CH$_3$ (b)	HOMO	LUMO
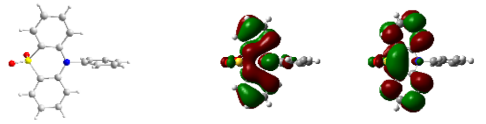			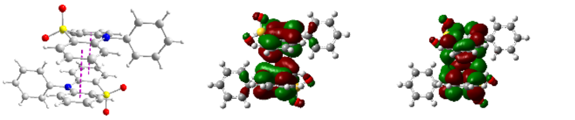		
CS-H	HOMO	LUMO	CS-H	HOMO	HOMO
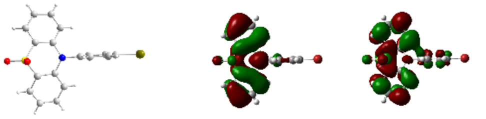			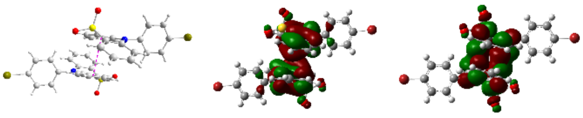		
CS-Br	HOMO	LUMO	CS-Br	HOMO	LUMO
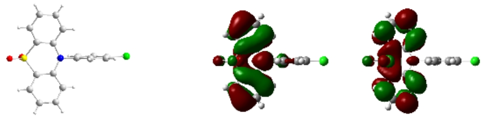			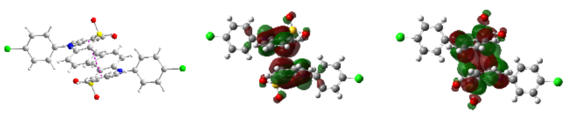		
CS-Cl	HOMO	LUMO	CS-Cl	HOMO	LUMO
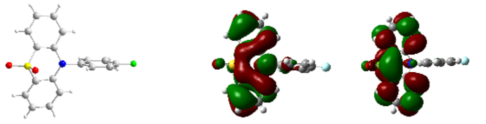			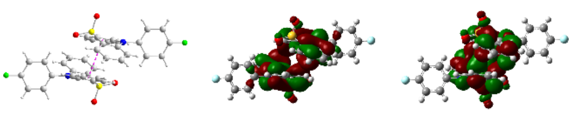		
CS-F	HOMO	LUMO	CS-F	HOMO	LUMO
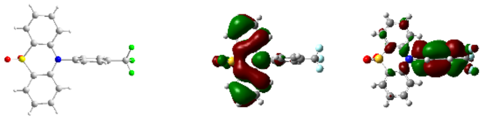			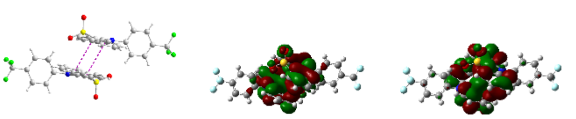		
CS-CF$_3$(i)	HOMO	LUMO	CS-CF$_3$(i)	HOMO	LUMO
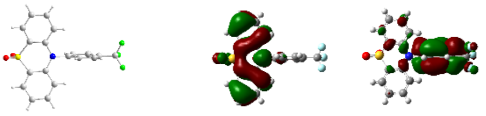			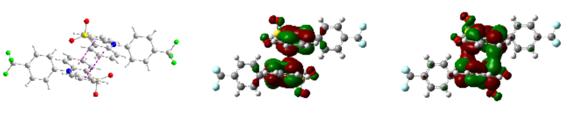		
CS-CF$_3$ (p)	HOMO	LUMO	CS-CF$_3$ (p)	HOMO	LUMO

Supplementary Table 4 The energy levels and gaps of triplet states for **CS-CH₃O**, **CS-CH₃**, **CS-H**, **CS-Br**, **CS-Cl** and **CS-F,** of which T_1' represents the excited triplet states of the central monomer molecule in crystal from QM/MM method, T_1 represents the excited triplet states of isolated molecule selected from the crystal from TD-DFT calculation and T_1* represents the excited triplet states of coupled units selected from the crystal from TD-DFT calculation.

Compound	$CS-CH_3O$	$CS-CH_3$(a)	$CS-CH_3$(b)	CS-H	CS-Br	CS-Cl	CS-F
T_1'-S_0 (eV)	3.8449	3.8839	3.8250	3.8447	3.9083	3.9738	3.9620
T_1-S_0(eV)	3.8472	3.8845	3.8256	3.8446	3.9098	3.9760	3.9653
T_1*-S_0 (eV)	3.8194	3.8297	3.7683	3.8035	3.8233	3.8594	3.8467
T_1-T_1*(eV)	0.0281	0.0548	0.0573	0.0411	0.0865	0.1166	0.1186

Supplementary Table 5 The energy levels of **$CS-CH_3O$**, **$CS-CH_3$**, **CS-H**, **CS-Br**, **CS-Cl** and **CS-F** in gas state from TD-DFT calculation.

Compound	$CS-CH_3O$	$CS-CH_3$	CS-H	CS-Br	CS-Cl	CS-F
S_1-S_0 (eV)	4.6516	4.6518	4.6575	4.6797	4.6792	4.6729
T_1-S_0(eV)	3.8867	3.8887	3.8918	3.9116	3.9110	3.9065
T_2-S_0 (eV)	3.9505	3.9508	3.9566	3.9803	3.9797	3.9724
T_3-S_0 (eV)	4.2018	4.2809	4.2809	4.2290	4.2506	4.2793
T_4-S_0(eV)	4.2816	4.3251	4.3659	4.2810	4.2802	4.3649
T_5-S_0(eV)	4.3584	4.3633	4.3935	4.3713	4.3722	4.3720
T_6-S_0(eV)	4.5692	4.6897	4.6898	4.6917	4.6917	4.6914
T_7-S_0(eV)	4.6892	4.8345	4.8350	4.8373	4.8370	4.8339
T_8-S_0(eV)	4.8388	4.9573	5.0169	4.8435	4.8371	4.8378
T_9-S_0(eV)	4.9425	4.9816	5.0434	4.8648	4.8915	5.0206
T_{10}-S_0(eV)	5.2766	5.0994	5.0669	4.9453	4.9636	5.1520

Supplementary Table 6 The energy levels of **$CS-CH_3O$**, **$CS-CH_3$**, **CS-H**, **CS-Br**, **CS-Cl** and **CS-F** in crystal state from QM/MM method.

Compound	$CS-CH_3O$	$CS-CH_3$(a)	$CS-CH_3$(b)	CS-H	CS-Br	CS-Cl	CS-F
S_1-S_0 (eV)	4.6125	4.6209	4.5798	4.6119	4.6730	4.6990	4.6862
T_1-S_0(eV)	3.8449	3.8839	3.8250	3.8447	3.9083	3.9738	3.9620
T_2-S_0 (eV)	3.9100	3.9223	3.8847	3.9083	3.9661	3.9912	3.9792
T_3-S_0 (eV)	4.2386	4.2568	4.2482	4.2695	4.2467	4.3076	4.3007
T_4-S_0 (eV)	4.2711	4.3155	4.2774	4.2890	4.2621	4.3651	4.3961
T_5-S_0 (eV)	4.3028	4.3511	4.3577	4.3947	4.3577	4.3984	4.4908
T_6-S_0 (eV)	4.5188	4.6369	4.6379	4.6183	4.6914	4.7024	4.6913
T_7-S_0 (eV)	4.6510	4.8033	4.8083	4.8002	4.8332	4.8611	4.8547
T_8-S_0 (eV)	4.8339	4.8965	4.9306	4.9756	4.8713	4.9576	4.9231
T_9-S_0 (eV)	4.9407	4.9590	4.9762	5.0605	4.8735	4.9629	5.1209
T_{10}-S_0 (eV)	5.2355	5.3167	5.1906	5.1637	4.9929	5.0533	5.2299

Supplementary Table 7 Difference Mulliken charge distribution and electrostatic potential (ESP) analysis of isolated **CS-CH$_3$O**, **CS-CH$_3$**, **CS-H**, **CS-Br**, **CS-Cl**, **CS-F**, **CS-CF$_3$** and their corresponding coupled units. The atomic charges range from -1.157 to 1.157, red indicates negative charge and green is positive charge. The potential energy range is -0.015 to 0.015 H q^{-1} for all surfaces shown, red indicates areas with dense electron density, yellow second, while blue areas suggest less electron density.

Single		Coupled Units	
charge	electrostatic potential	charge	electrostatic potential
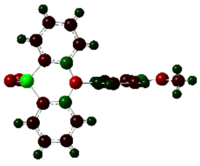 CS-CH$_3$O	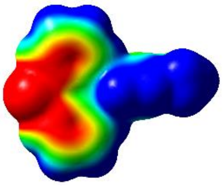	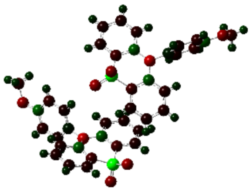	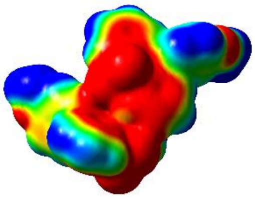
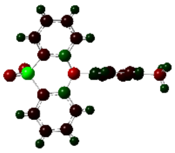 CS-CH$_3$(a)	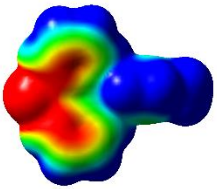	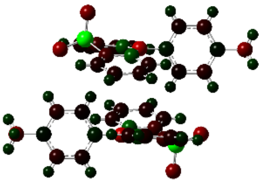	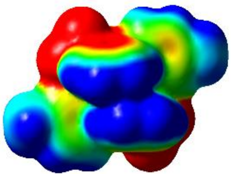
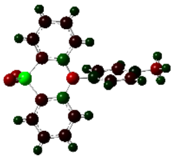 CS-CH$_3$ (b)	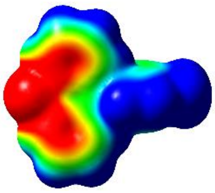	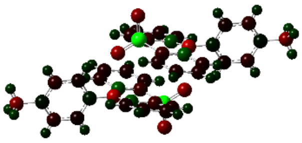	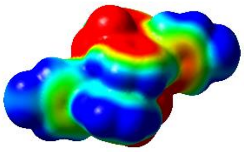
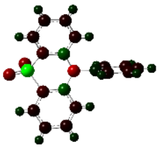 CS-H	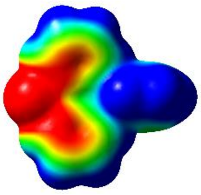	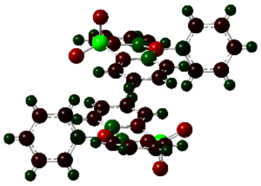	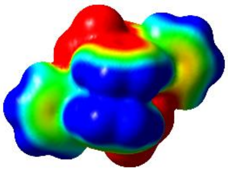
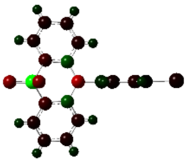 CS-Br	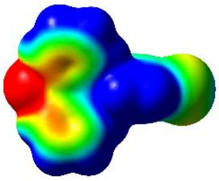	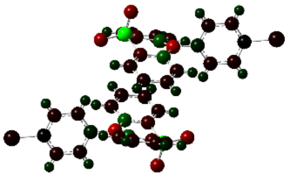	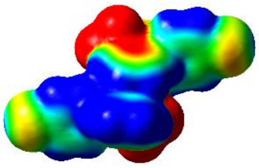
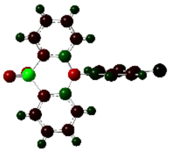 CS-Cl	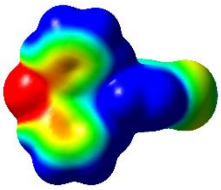	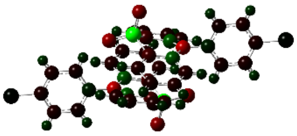	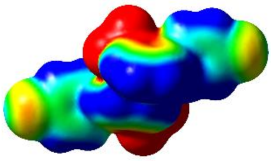
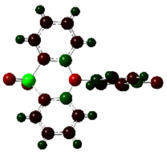 CS-F	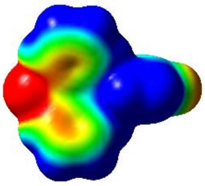	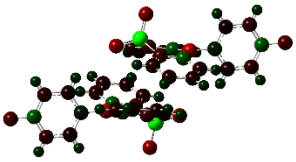	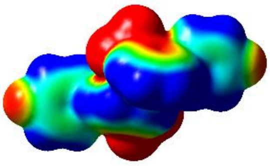

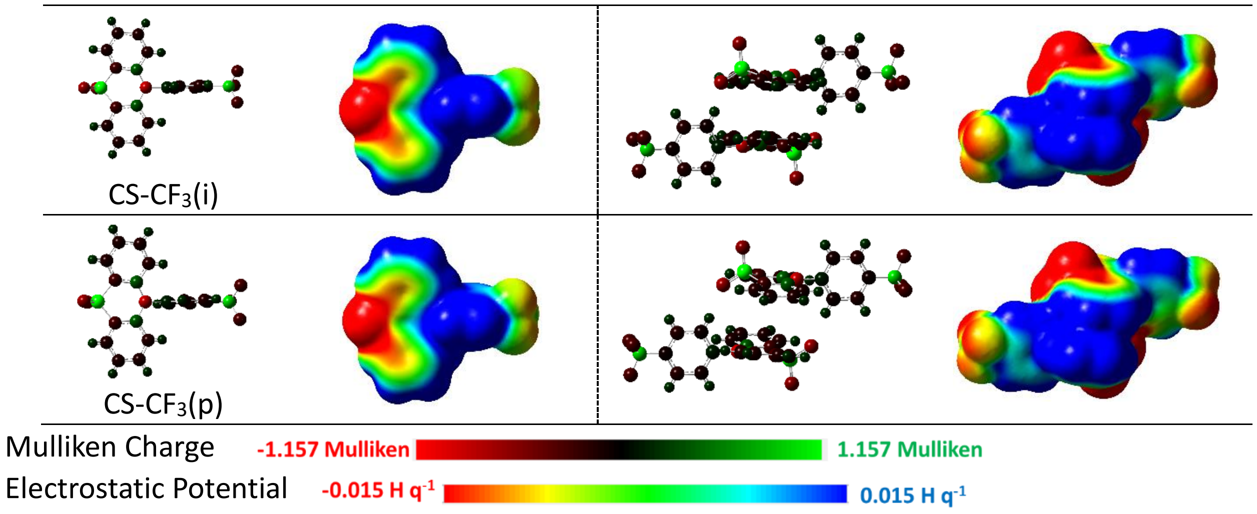

Supplementary Table 8 Structure data of **$CS\text{-}CF_3$** single crystals before (initial state) and after (phosphorescence state) the five minutes 365 UV-irradiation.

Type	initial state	phosphorescence state
Formula	$C_{19}H_{12}F_3NO_2S$	$C_{19}H_{12}F_3NO_2S$
Wavelength (Å)	1.54184	1.54184
Space Group	P -1	P -1
Cell Lengths (Å)	a=8.0146(8)<br>b=9.1619(9)<br>c=11.7227(11)	a=8.0303(6)<br>b=9.1720(5)<br>c=11.6958(7)
Cell Angles (°)	α=96.919(8)<br>β=96.067(8)<br>γ=109.322(9)	α=96.971(5)<br>β=96.058(6)<br>γ=109.220(6)
Cell Volume ($Å^3$)	796.60(14)	797.63(10)
Z	2	2
Density (g/cm^3)	1.565	1.563
F(000)	384.0	384.0
h_{max}, k_{max}, l_{max}	9, 10, 13	9,10,13
CCDC number	1519670	1515625

Supplementary Discussion

Understanding molecular aggregation

The self-association of luminogens in solution or in the solid state is a frequently encountered phenomenon for its significant effects on their photophysical property. The aggregates exhibit notable changes in the absorption band as compared to the monomeric species, mainly two kinds of changes—J aggregate with the bathochromic-shifted absorption and H aggregate with hypochromatic-shifted absorption in aggregates. Thus we collected two molecules in our system to study their aggregate mode—One is **$CS\text{-}CH_3O$** with shortest RTP lifetime of 88 ms and the other is **CS-F** with longest RTP lifetime up to 410 ms. On the other hand, a classical RTP luminogen **DEOPh** with H-aggregation is studied as the comparison.

From the experimental data in Supplementary Figure 10, it is clear that **CS-CH_3O** and **CS-F** are all J-aggregates for the bathochromic-shifted absorption from solution to aggregation, while **DEOPh** is H-aggregate for the hypochromatic-shifted absorption from solution to aggregation. Thus, it is the π-π stacking, rather than H-aggregation should be mainly responsible for the RTP effect in our system. On the other hand, as the absorption of both compounds CS-CH_3O and CS-F are equally affected by aggregation, but show marked different RTP responses in their crystal forms, this indicates that the aggregates observed in solution are not responsible for the different RTP response in the crystals.

Supplementary References

1 An, Z. *et al.* Stabilizing triplet excited states for ultralong organic phosphorescence. *Nat. Mater.* **14**, 685–690 (2015).

2 Zhao, W. et al. Rational Molecular Design for Achieving Persistent and Efficient Pure Organic Room-Temperature Phosphorescence. *Chem* **1**, 592–602 (2016).

3 Li, C. *et al.* Reversible Luminescence Switching of an Organic Solid: Controllable On–Off Persistent Room Temperature Phosphorescence and Stimulated Multiple Fluorescence Conversion. *Adv. Optical Mater.* **3**, 1184–1190 (2015).

4 Mao, Z. *et al.* Linearly tunable emission colors obtained from a fluorescent-phosphorescent dual-emission compound by mechanical stimuli. *Angew. Chem.* **127**, 6368–6371 (2015).

5 Gong, Y. *et al.* Achieving Persistent Room Temperature Phosphorescence and Remarkable Mechanochromism from Pure Organic Luminogens. *Adv. Mater.* **27**, 6195–6201 (2015).

6 Xue, P. *et al.* Luminescence switching of a persistent room-temperature phosphorescent pure organic molecule in response to external stimuli. *Chem. Commun.* **51**, 10381–10384 (2015).

7 Yang, Z. *et al.* Intermolecular Electronic Coupling of Organic Units for Efficient Persistent Room-Temperature Phosphorescence. *Angew. Chem.* **128**, 2221–2225 (2016).

8 Xu, B. *et al.* White-light emission from a single heavy-atom-free molecule with room temperature phosphorescence, mechanochromism and thermochromism. *Chem. Sci.* **8**, 1909–1914 (2017).

9 Gong, Y. *et al.* Crystallization-induced dual emission from metal- and heavy atom-free aromatic acids and esters. *Chem. Sci.* **6**, 4438–4444 (2015).
