# Supplementary material for: The influence of the molecular packing on the room temperature phosphorescence of purely organic luminogens
Source: Nat Commun. 2018 Feb 26;9:840. doi: 10.1038/s41467-018-03236-6 (PMC5826932; doi:10.1038/s41467-018-03236-6)
Supplement: Supplementary file 3 — Description of Additional Supplementary Files [file 41467_2018_3236_MOESM3_ESM.pdf]

Descriptions of Additional Supplementary Files

File Name: Supplementary Movie 1
Description: The video of CS-CF3 crystals after turning off the UV lamp immediately.

File Name: Supplementary Movie 2
Description: The video of CS-CF3 crystals after irridiating by a UV lamp of 365 nm for five minutes and then turning off the UV lamp.

File Name: Supplementary Dataset 1
Description: The collection of the crystal cif files.

File Name: Supplementary Dataset 2
Description: The collection of the crystal checkcif files.
